# Photocatalytic doping of organic semiconductors

**DOI:** 10.1038/s41586-024-07400-5

**Published:** 2024-05-15

**Authors:** Wenlong Jin, Chi-Yuan Yang, Riccardo Pau, Qingqing Wang, Eelco K. Tekelenburg, Han-Yan Wu, Ziang Wu, Sang Young Jeong, Federico Pitzalis, Tiefeng Liu, Qiao He, Qifan Li, Jun-Da Huang, Renee Kroon, Martin Heeney, Han Young Woo, Andrea Mura, Alessandro Motta, Antonio Facchetti, Mats Fahlman, Maria Antonietta Loi, Simone Fabiano

**Affiliations:** 1 Laboratory of Organic Electronics, Department of Science and Technology, Linköping University, Norrköping, Sweden; 2 n-Ink AB, Norrköping, Sweden; 3 Zernike Institute for Advanced Materials, University of Groningen, Groningen, The Netherlands; 4 Dipartimento di Fisica, Università degli Studi di Cagliari, Monserrato, Italy; 5 Department of Chemistry, College of Science, Korea University, Seoul, Republic of Korea; 6 Wallenberg Initiative Materials Science for Sustainability, Department of Science and Technology, Linköping University, Norrköping, Sweden; 7 Department of Chemistry and Centre for Processable Electronics, Imperial College London, London, UK; 8 Dipartimento di Scienze Chimiche, Università di Roma “La Sapienza” and INSTM, UdR Roma, Rome, Italy; 9 School of Materials Science and Engineering, Georgia Institute of Technology, Atlanta, GA USA

**Keywords:** Electronic devices, Photocatalysis, Electronic materials

## Abstract

Chemical doping is an important approach to manipulating charge-carrier concentration and transport in organic semiconductors (OSCs)^1–3^ and ultimately enhances device performance^4–7^. However, conventional doping strategies often rely on the use of highly reactive (strong) dopants^8–10^, which are consumed during the doping process. Achieving efficient doping with weak and/or widely accessible dopants under mild conditions remains a considerable challenge. Here, we report a previously undescribed concept for the photocatalytic doping of OSCs that uses air as a weak oxidant (p-dopant) and operates at room temperature. This is a general approach that can be applied to various OSCs and photocatalysts, yielding electrical conductivities that exceed 3,000 S cm^–1^. We also demonstrate the successful photocatalytic reduction (n-doping) and simultaneous p-doping and n-doping of OSCs in which the organic salt used to maintain charge neutrality is the only chemical consumed. Our photocatalytic doping method offers great potential for advancing OSC doping and developing next-generation organic electronic devices.

## Abstract (web summary)

A previously undescribed photocatalytic approach enables the effective p-type and n-type doping of organic semiconductors at room temperature using only widely available weak dopants such as oxygen and triethylamine.

## Main

Chemical doping has a pivotal role in optimizing the performance of various OSC-based devices, including light-emitting diodes^11^, photovoltaics^12,13^, thermoelectrics^14,15^, field-effect transistors^16^ and electrochemical devices^17,18^. Although numerous doping methods exist that can achieve efficient chemical doping, they typically rely on the use of strong oxidizing and reducing agents (dopants) to alter the redox state of the OSC through direct electron-transfer processes^19–27^. However, the high chemical reactivity of these strong dopants may produce by-products and render them less stable, particularly for n-dopants^5,10,14,15^, presenting a challenge to their widespread adoption. Furthermore, some of the most-studied dopants are synthesized ad hoc and are expensive. The use of widely available, inexpensive and weak or inefficient dopants could address these issues, but they often require either thermal^28–31^ or radiation^32–35^ activation or metal-catalysed bond cleavage^36^ to activate them. Moreover, there is currently no dopant that is capable of carrying out both the oxidation (p-doping) and reduction (n-doping) of OSCs. Therefore, developing a doping strategy that uses widely available dopants is crucial to enhance compatibility with semiconductor devices.

Photocatalysts (PCs) function as electron shuttles, oxidizing or reducing aromatic compounds on the basis of the presence of sacrificial weak oxidants^37,38^ or reductants^39,40^. Owing to their remarkable selectivity and efficiency in facilitating redox-based reactions, PCs are extensively used in organic synthesis^41–44^. Thus, intriguing questions arise regarding whether PCs can mediate the oxidation (p-doping) or reduction (n-doping) of OSCs, the efficiency and generalizability of this process, and its potential technological implications.

**Fig. 1:**
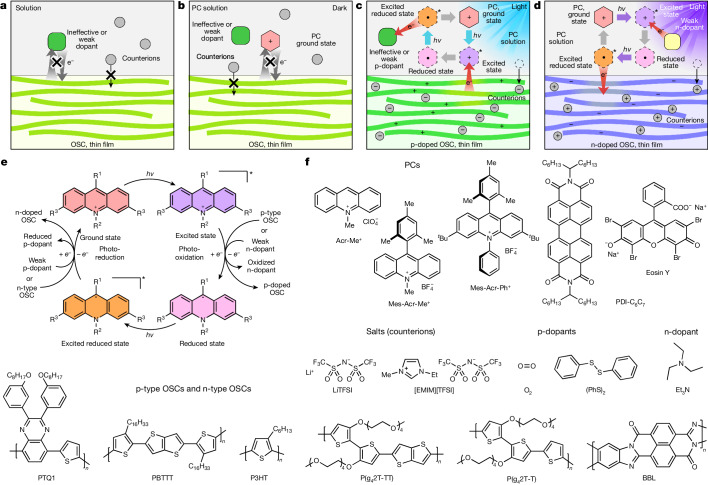
Photocatalytic doping concept. **a**–**d**, Schematics of the photocatalytic doping process: weak dopants cannot oxidize or reduce the OSCs (**a**); PCs in the ground state cannot oxidize or reduce the OSCs (**b**); PCs in the excited state can oxidize or reduce the OSCs and be regenerated by the weak dopants (**c** and **d**). This photocatalytic doping process occurs in thin films and ensures that the molecular packing of the OSCs is preserved after doping. **e**,**f**, Proposed photocatalytic oxidation or p-doping (right) and reduction or n-doping (left) cycle (**e**); chemical structures of the PCs, weak dopants, salts (counterions) and OSCs (**f**).

**Extended Data Fig. 1:**
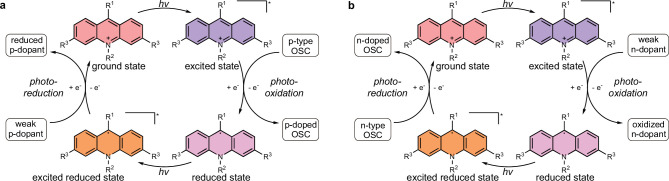
Photocatalytic doping concept. **a**, Photocatalytic oxidation/p-doping cycle. The photocatalyst in the ground state is photoactivated to its excited state and converted to its reduced state upon oxidation of the organic semiconductor. The photocatalyst in the reduced state is activated to its excited reduced state and then regenerated by the weak p-dopant. **b**, Photocatalytic reduction/n-doping cycle. The photocatalyst is first photoactivated to its excited state and can oxidize the weak n-dopant. Upon oxidation of the latter, the photocatalyst is converted to its reduced state and then photoactivated to obtain an excited reduced state capable of reducing the organic semiconductor.

Here, to demonstrate the general concept, we explore the photocatalytic doping process in quaternary systems comprising a dopant, a PC that was primarily an acridinium derivative, an organic salt and one of various π-conjugated p-type or n-type OSC polymers. We show that neither the dopant nor the PC in the ground state can extract or donate electrons from or to the OSCs (Fig. 1a,b). However, when photoexcitation occurs, the PC can oxidize or reduce the OSC and be regenerated by the dopant (Fig. 1c–e). Extended Data Fig. 1 provides a breakdown of the photocatalytic p-doping and n-doping cycles. Acridinium-based PCs are used because of their commercial availability, air stability and strong oxidizing or reducing character in their excited or radical reduced state^40,45,46^. We chose three different acridinium derivatives to carry out the photocatalytic doping study: Acr-Me^+^ (10-methylacridinium perchlorate), Mes-Acr-Me^+^ (9-mesityl-10-methylacridinium tetrafluoroborate) and Mes-Acr-Ph^+^ (9-mesityl-3,6-di-*tert*-butyl-10-phenylacridinium tetrafluoroborate). We compared their activity with that of other air-stable organic PCs, such as perylene diimide^39^ (PDI-C_6_C_7_) and eosin Y^47,48^ (2′,4′,5′,7′-tetrabromofluorescein disodium salt) (Fig. 1f). Salts such as LiTFSI and [EMIM][TFSI] are used as redox-inert counterions to stabilize charges on the doped conjugated polymer backbones^27^. We used oxidants (p-dopants) that are typically weak or inefficient, such as dioxygen (O_2_) in the air or diphenyl disulfide^49^ ((PhS)_2_), and weak reductants (n-dopants), such as triethylamine (Et_3_N), to regenerate the PCs.

## Photocatalytic p-doping of PBTTT

**Fig. 2:**
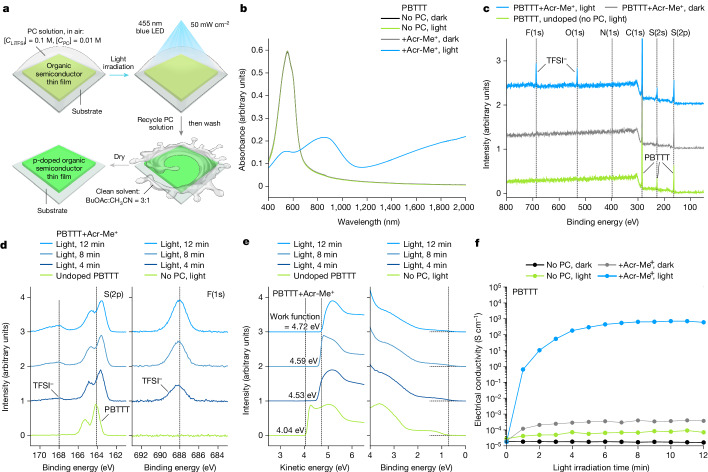
Photocatalytic p-doping of PBTTT. **a**, Schematic of the photocatalytic p-doping method: the OSC film is immersed in the PC solution, which also contains TFSI^–^ counterions, and irradiated with light in the presence of O_2_, which acts as the weak p-dopant. The PC solution is recovered and the OSC film is washed with clean solvent and dried in N_2_. [*C*_LiTFSI_] and [*C*_PC_] are the concentrations of the LiTFSI and PC solutions, respectively. **b**, Absorption spectra of undoped and photocatalytically doped PBTTT films. Photocatalytic doping requires both PC and light. **c**, XPS analysis of undoped and photocatalytically doped PBTTT films reveals a distinct TFSI^–^ signal in the photocatalytically doped samples. **d**, S(2p) and F(1s) XPS analysis of undoped and photocatalytically doped PBTTT films. The TFSI^–^ signal increases with light irradiation time. **e**, Ultraviolet photoelectron spectroscopy of undoped and photocatalytically doped PBTTT films. The work function of photocatalytically doped PBTTT films increases with light irradiation time. **f**, Electrical conductivity of undoped and photocatalytically doped PBTTT films demonstrates that photocatalytic doping can happen only when both PC and light are present. Points, mean; error bars, s.d. (not visible); *n* = 10 independent samples. Source Data

The photocatalytic p-doping methodology is straightforward and efficient, as demonstrated in Fig. 2 and Supplementary Video 1 for the case-study oxidation of PBTTT by Acr-Me^+^. Acr-Me^+^ (0.01 M) was dissolved in a mixture of *n*-butyl acetate and acetonitrile, together with the salt LiTFSI (0.1 M). This solvent mixture was chosen for its orthogonality to PBTTT and the good solubility of Acr-Me^+^ and LiTFSI, which show no substantial interactions in solution (Supplementary Figs. 1 and 2). PBTTT thin films were immersed in the Acr-Me:LiTFSI solution and irradiated with a 455 nm blue light for up to 12 min to photoactivate Acr-Me^+^ to its excited state (Acr-Me^+*^; ref. ^37^) in the presence of O_2_ (in air), which acts as the weak p-dopant. The p-doped PBTTT films were then removed from the PC solution, washed and dried, as depicted in Fig. 2a, and the Acr-Me:LiTFSI solution was recovered and could be reused multiple times (Supplementary Fig. 3a). The ultraviolet–visible–near-infrared absorption spectra of PBTTT films show that O_2_ cannot dope PBTTT in the absence of Acr-Me^+^ or with Acr-Me^+^ in the dark (Fig. 2b), but a strong polaronic absorption extending in the near-infrared region was observed upon blue light irradiation.

**Extended Data Fig. 2:**
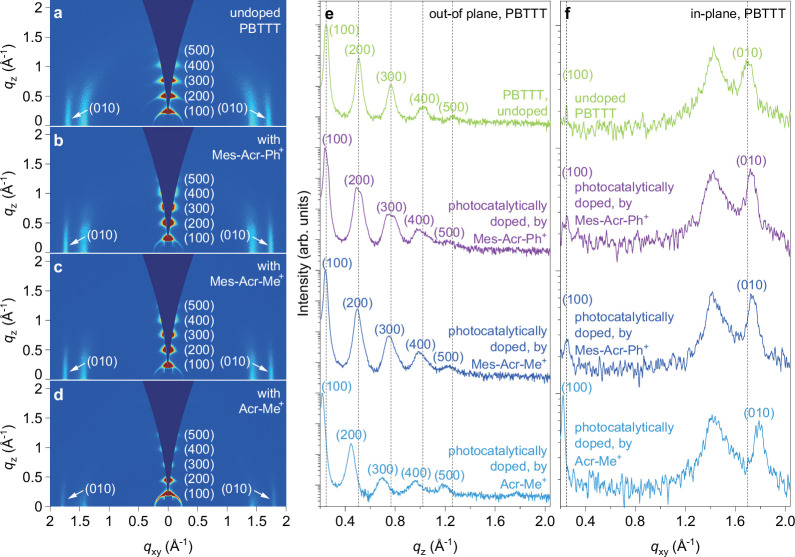
GIWAXS analysis of PBTTT. **a-d**, 2D GIWAXS patterns of undoped PBTTT (**a**) and doped PBTTT thin films photocatalyzed by different photocatalysts Mes-Acr-Ph^+^ (**b**), Mes-Acr-Me^+^ (**c**), and Acr-Me^+^ (**d**). **e-f**, Out-of-plane (**e**) and in-plane (**f**) GIWAXS line cuts of undoped and doped PBTTT thin films.

**Extended Data Fig. 3:**
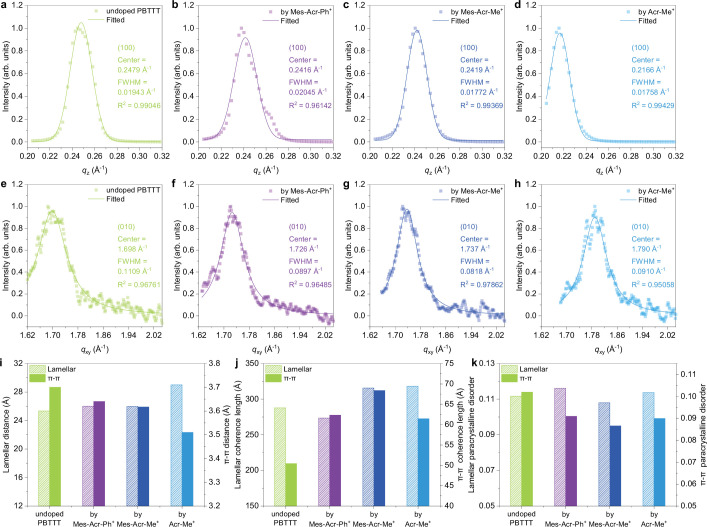
Lamellar packing and π-π stacking of PBTTT. **a-d**, Lamellar packing diffraction peak analysis of undoped PBTTT (**a**) and doped PBTTT thin films photocatalyzed by Mes-Acr-Ph^+^ (**b**), Mes-Acr-Me^+^ (**c**), and Acr-Me^+^ (**d**). **e-h**, π-π stacking diffraction peak analysis of undoped PBTTT (**e**) and doped PBTTT thin films photocatalyzed by Mes-Acr-Ph^+^ (**f**), Mes-Acr-Me^+^ (**g**), and Acr-Me^+^ (**h**). **i-k**, Packing distances (**i**), coherence length (**j**), and paracrystalline disorder (**k**) of lamellar packing and π-π stacking of PBTTT. Photocatalyzed doped PBTTT shows longer lamellar packing distance, shorter π-π stacking distance, and more regular π-π stacking than undoped PBTTT.

X-ray photoelectron spectroscopy (XPS) shows that TFSI^–^ anions do not penetrate the PBTTT layer without Acr-Me^+^ or with Acr-Me^+^ in the dark. However, when irradiated with light, strong fluorine F(1s) and oxygen O(1s) XPS core level signals originating from TFSI^–^ are observed (Fig. 2c). Further analysis of the sulfur S(2p), F(1s) and nitrogen N(1s) XPS core level peaks (Fig. 2d and Supplementary Fig. 4) indicates an increase in TFSI^–^ anion content with increasing irradiation time, leading to a positively charged PBTTT polymer backbone. Grazing-incidence wide-angle X-ray scattering (GIWAXS) measurements reveal that the penetration of TFSI^–^ anions does not disrupt the orientation of the PBTTT chains but instead leads to a reduction in the π–π stacking distance and an increase in the lamellar packing distance, indicating that TFSI^–^ anions are located mainly in the side-chain region (Extended Data Figs. 2, and 3 and Supplementary Figs. 5–7). This result is consistent with the oxidation of PBTTT^19^. Furthermore, ultraviolet photoelectron spectroscopy (UPS) analysis reveals a partial depopulation of the PBTTT frontier occupied density of states and a substantial increase in work function following light irradiation (Fig. 2e and Supplementary Fig. 8).

**Extended Data Fig. 4:**
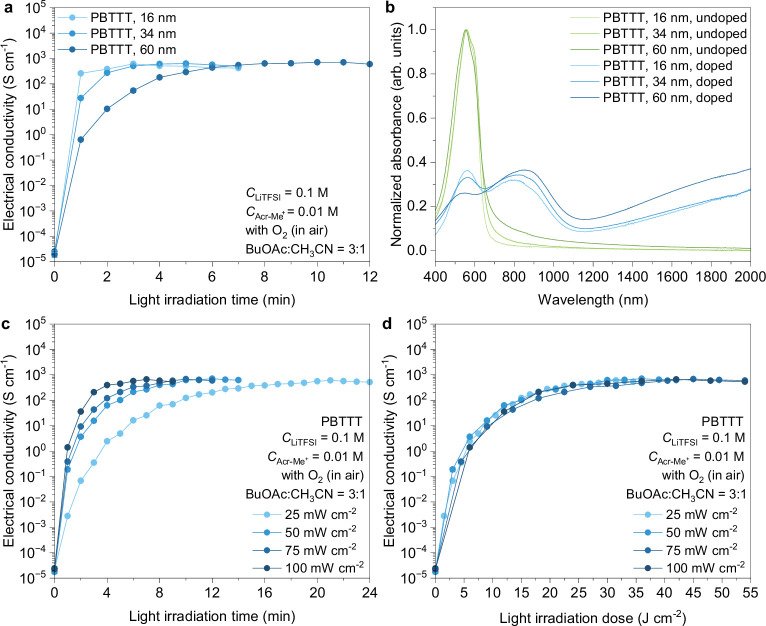
Effect of thickness and light irradiation power. **a**, Electrical conductivity of doped PBTTT (photocatalyzed by Acr-Me^+^) films with various thicknesses. Points, mean; error bars, s.d. (not visible); n = 10 independent samples. **b**, Absorption spectra of undoped and doped PBTTT films with various thicknesses. The doped PBTTT shows similar maximum conductivity and polaron absorption intensity regardless of the film thicknesses, suggesting that photocatalytic doping is a bulk doping process. **c-d**, Electrical conductivity of doped PBTTT (photocatalyzed by Acr-Me^+^) films under different light exposure power, plotted as a function of light irradiation time (**c**) and light irradiation dose (**d**). Points, mean; error bars, s.d. (not visible); n = 10 independent samples.

The electrical conductivity of PBTTT greatly increases from 10^−5^ S cm^−1^ to more than 700 S cm^−1^ after 10 min of light irradiation (Fig. 2f) and remains stable after 30 days of storage (Supplementary Fig. 3b). By contrast, no major changes in electrical conductivity are observed either without Acr-Me^+^ or with Acr-Me^+^ in the dark. Notably, the observed conductivity values are typical for chemically p-doped PBTTT^27^. We also noticed that PBTTT films, spanning a thickness range of 16 nm to 60 nm, exhibited similar maximum conductivity and polaronic absorption intensity, with the thinner films being doped faster (Extended Data Fig. 4a,b). These results indicate that doping occurs throughout the entire bulk of the OSC layer. Moreover, the degree of doping depends on the light irradiation dose (Extended Data Fig. 4c,d) and wavelength (Supplementary Fig. 9).

## Mechanism of photocatalytic p-doping

**Fig. 3:**
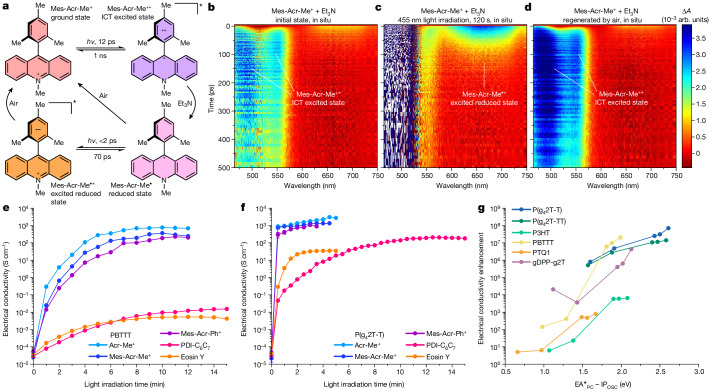
Mechanism and generality of the photocatalytic p-doping process. **a**, Schematic diagram of the transition of the ground state, excited state, reduced state and excited reduced state of Mes-Acr-Me^+^ in the presence of the optically transparent Et_3_N in 455 nm irradiation, followed by regeneration in air. **b**–**d**, False-colour plots of the in situ transient absorption spectra of Mes-Acr-Me^+^ + 10 equivalents Et_3_N, as a function of detector wavelength and delay time: initial state in N_2_ atmosphere (**b**); after 455 nm light irradiation for 120 s in N_2_ (**c**); and regeneration in air (**d**). Two strong photoinduced absorption peaks (477 nm and 551 nm, **b** and **d**) indicate the formation of the excited state of the PC (Mes-Acr-Me^+*^). The photoinduced absorption peak at 658 nm (**c**) indicates the formation of the PC excited reduced state (Mes-Acr-Me^•*^). **e**,**f**, Electrical conductivity of PBTTT (**e**) and P(g_4_2T-T) (**f**) photocatalytically doped by different PCs in air. The photocatalytic p-doping conditions are the same as in Fig. 2a. Points, mean; error bars, s.d. (not visible); *n* = 10 independent samples. **g**, Electrical conductivity enhancement of OSCs (compared with the undoped films) plotted against the energy barrier of single-electron transfer from OSCs to excited PCs. Source Data

**Extended Data Fig. 5:**
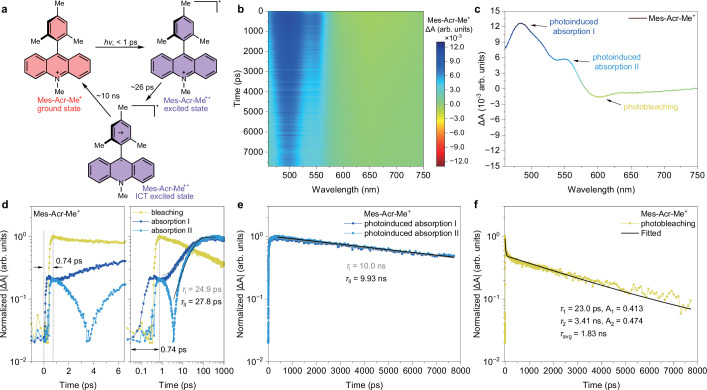
TAS of Mes-Acr-Me^+^. **a**, Schematic diagram of the transitions from the ground state to the excited state and the intramolecular charge-transfer (ICT) excited state of Mes-Acr-Me^+^, under 390 nm laser pump pulse. **b**, False color representation of the transient absorption spectra of Mes-Acr-Me^+^ solution (0.01 M, with 0.1 M of LiTFSI, in BuOAc:CH_3_CN = 3:1) as a function of delay time and detector wavelength. **c**, Transient absorption spectra of Mes-Acr-Me^+^ showing the photobleaching (peak center 601 nm) and two significant photoinduced absorption peaks (I, 484 nm; II, 549 nm). **d**, Time-dependent photobleaching and photoinduced absorption of Mes-Acr-Me^+^, showing immediate raising (< 1 ps, limited by instrumental time resolution) for photobleaching, and immediate raising, slow falling, and slower again raising (raising time ~ 26 ps), indicating the formation of the ICT excited state. **e**, Time-dependent photoinduced absorption of Mes-Acr-Me^+^, showing a long excited lifetime of 10 ns. **f**, Time-dependent photobleaching of Mes-Acr-Me^+^, showing a photobleaching time constant of 1.8 ns.

**Extended Data Fig. 6:**
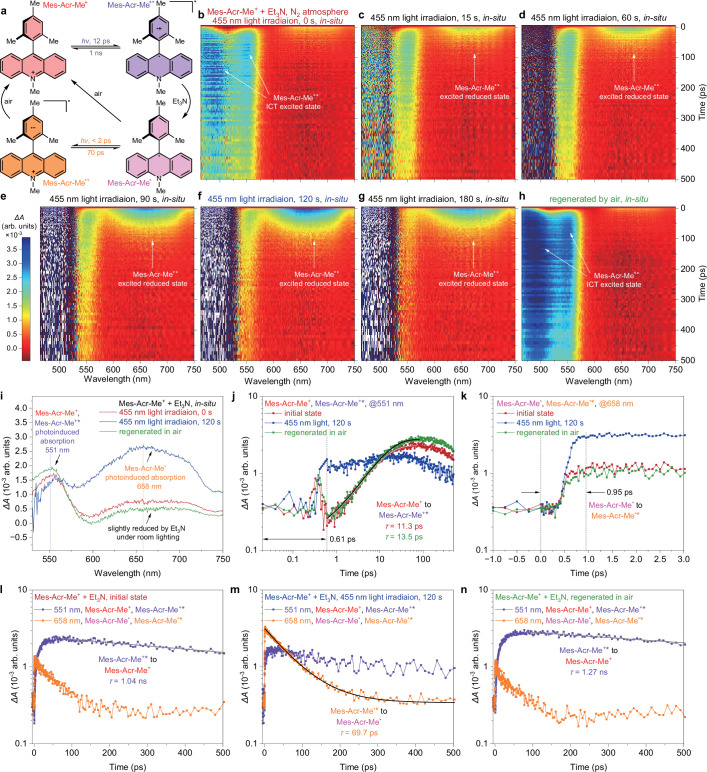
*In-situ* TAS of Mes-Acr-Me^+^/Et_3_N. **a**, Schematic diagram of the transition from the ground state to the excited state to the reduced state and excited reduced state of Mes-Acr-Me^+^. **b-h**, False color representation of the *in-situ* transient absorption spectra of Mes-Acr-Me^+^ solution (0.01 M, with 0.1 M of Et_3_N, 0.1 M of LiTFSI, in BuOAc:CH_3_CN = 3:1) as a function of delay time and detector wavelength, with different 455 nm light irradiation time: initial state 0 s (**b**), 15 s (**c**), 60 s (**d**), 90 s (**e**), 120 s (**f**), 180 s (**g**), and regenerated in air (**h**). The transient absorption spectra reveal two strong photoinduced absorption peaks (477 nm and 551 nm) in the initial and regenerated Mes-Acr-Me^+^, showing the ICT excited state (Mes-Acr-Me^+*^). The new photoinduced absorption peak at 658 nm is ascribed to the excited reduced state (Mes-Acr-Me^•*^). The transient absorption spectra recovered almost completely to the initial state after exposure to air, suggesting the regeneration of Mes-Acr-Me^+^ by air oxidation. **i**, *In-situ* transient absorption spectra of the Mes-Acr-Me^+^/Et_3_N solution in the initial state, after 455 nm light irradiation time of 120 s, and regenerated in air. The transient absorption spectra present the formation of a reduced state (Mes-Acr-Me^•^) and excited reduced state (Mes-Acr-Me^•*^), and regeneration to Mes-Acr-Me^+^ by air oxidation. **j-k**, Time-dependent photoinduced absorption, showing similar features in the initial and regenerated states of Mes-Acr-Me^+^/Et_3_N solution, indicating the same ICT excited state formation (Mes-Acr-Me^+*^). **l-n**, Time-dependent photoinduced absorption of Mes-Acr-Me^+^/Et_3_N solution after irradiation at 455 nm for 0 s (**l**) and 120 s (**m**), and regeneration in air (**n**), showing that the excited lifetime of Mes-Acr-Me^+^ is ~1 ns (in the presence of 10 eq Et_3_N), and excited lifetime of Mes-Acr-Me^•^ is 70 ps.

**Extended Data Fig. 7:**
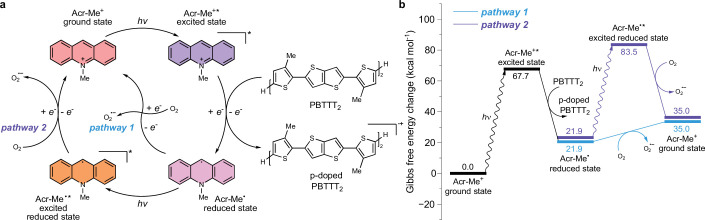
Photocatalytic p-doping mechanisms. **a**, Photocatalytic p-doping cycle of PBTTT in the presence of Acr-Me^+^ and O_2_. **b**, Corresponding DFT-calculated Gibbs free energy profiles.

To understand the charge-transfer mechanism involved in the photocatalytic p-doping process, we performed transient absorption spectroscopy and related photoluminescence and absorption spectroscopy experiments. Analysis of the Acr-Me^+^ and PBTTT excited-state lifetimes (Supplementary Figs. 10–17) and quantitative fluorescence-quenching experiments (Supplementary Figs. 18–20) indicates that the photo-induced electron transfer (PET) process is probably dominated by the PC excited state, with electrons predominately transferring from the OSC ground state to the PC excited state. We used Et_3_N as an optically transparent reductant^39^ to study the PET process in situ (Fig. 3a–d). Mes-Acr-Me^+^ and Mes-Acr-Ph^+^ were selected as representative PCs because of their excellent stability in the presence of Et_3_N (Supplementary Fig. 21). PET between Mes-Acr-Me^+*^ (or Mes-Acr-Ph^+*^) and Et_3_N induces the formation of reduced Mes-Acr-Me^*•*^ (or Mes-Acr-Ph^*•*^)^40,46^, as revealed by the absorption spectra shown in Supplementary Figs. 21 and 22. Subsequently, Mes-Acr-Me^*•*^ (or Mes-Acr-Ph^*•*^) is oxidized by O_2_ (ref. ^37^) (or (PhS)_2_; ref. ^49^), affording reduced species (Supplementary Figs. 23 and 24). In situ photoluminescence studies demonstrate the reduction and regeneration of Mes-Acr-Me^+^ and Mes-Acr-Ph^+^ (Supplementary Fig. 25). Moreover, in situ transient absorption spectra acquired during the reduction or regeneration process reveal that Mes-Acr-Me^+^ initially forms an intramolecular-charge-transfer excited state (Mes-Acr-Me^+*^)^38,50^ as a result of light irradiation (Fig. 3a,b and Extended Data Figs. 5 and 6). Subsequently, Mes-Acr-Me^+*^ transitions to the reduced Mes-Acr-Me^*•*^ in the presence of Et_3_N. A notable photoinduced absorption at 658 nm indicates the formation of an excited reduced state for Mes-Acr-Me after a laser pump pulse (Fig. 3c). Oxidation in air leads to Mes-Acr-Me^+^ being fully regenerated (Fig. 3d, Extended Data Fig. 6 and Supplementary Video 2). This result demonstrates that the PET process requires only the photoexcitation of PCs and that PCs can be regenerated in air, thereby closing the photocatalytic oxidation cycle. The Gibbs free-energy profiles computed using density functional theory (DFT) for the photocatalytic doping of PBTTT by Acr-Me^+^ in air indicate that both the reduced and excited reduced forms of Acr-Me can be oxidized by O_2_, with Acr-Me^•*^ being favoured (Extended Data Fig. 7).

## Generality of photocatalytic p-doping

**Extended Data Fig. 8:**
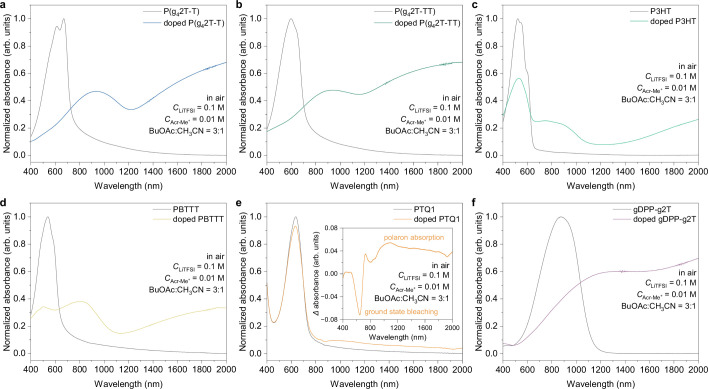
Generality of photocatalytic doping: absorption spectra. **a-f**, Absorption spectra of undoped and doped (photocatalyzed by Acr-Me^+^/air) P(g_4_2T-T) (**a**), P(g_4_2T-TT) (**b**), P3HT (**c**), PBTTT (**d**), PTQ1 (**e**), and gDPP-g2T (**f**) films.

Next, we investigated the generality of the photocatalytic p-doping process for a range of conjugated polymers with different ionization-potential values and hydrophilic or hydrophobic side chains (such as P(g_4_2T-T), P(g_4_2T-TT), gDPP-g2T, P3HT, PBTTT and PTQ1) and PCs with different electron affinity (EA) values, including Acr-Me^+^, Mes-Acr-Me^+^, Mes-Acr-Ph^+^, perylene diimide and eosin Y (Fig. 1d). The ionization potentials of the semiconducting polymers and the EA values of the PCs used here, as well as the EA values of conventional molecular p-dopants such as F_4_TCNQ, F_6_TCNNQ, magic blue, CN6-CP and NOPF_6_ used for comparative analysis, are reported in Supplementary Fig. 26. In the ground state, all PCs exhibit low EAs, ranging from 3.6 to 4.2 eV, which are insufficient to thermodynamically drive the p-doping of the OSCs with ionization potentials ranging from 4.3 to 5.3 eV. However, when photoactivated, the EAs of the PC in the excited state (EA^*^) are estimated to be in the range 5.9–6.9 eV, thereby enabling p-doping of the aforementioned OSCs. The absorption spectra of the photocatalytically doped polymer films demonstrate that all the semiconducting polymers investigated in this study, including the high-ionization-potential polymer PTQ1, can be photocatalytically doped by Acr-Me^+^ (Extended Data Fig. 8). These data indicate that Acr-Me^+^ serves as a strong oxidant, at least comparable in strength to Mo(tfd)_3_ (EA of around 5.5 eV)^51^, although the calculated EA^*^ indicates that its oxidizing capability is probably much stronger.

**Extended Data Fig. 9:**
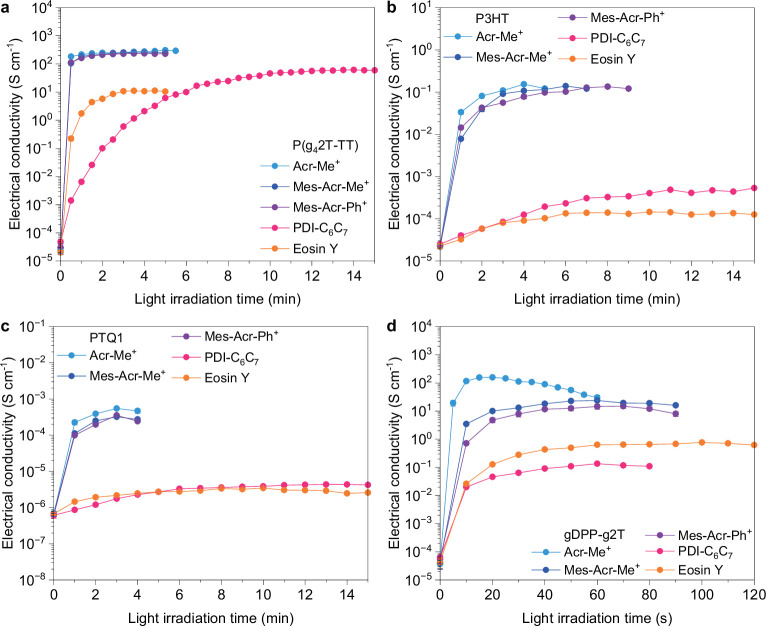
Generality of photocatalytic doping: electrical conductivity. **a-d**, Electrical conductivity of PTQ1 (**a**), P3HT (**b**), P(g_4_2T-TT) (**c**) and gDPP-g2T (**d**), the thin films were photocatalytically doped by different photocatalysts. Points, mean; error bars, s.d. (not visible); n = 10 independent samples.

The electrical conductivity of the photocatalytically doped polymeric thin films was investigated for the different PCs and as a function of the light-irradiation time (Fig. 3e,f, Extended Data Fig. 9 and Supplementary Table 1). P(g_4_2T-T), P(g_4_2T-TT), gDPP-g2T and PBTTT were doped by all five PCs in the presence of O_2_, with P(g_4_2T-T), which has an ionization potential of around 4.3 eV (ref. ^21^), exhibiting a high electrical conductivity of up to 3,000 S cm^−1^. Comparable results were obtained using (PhS)_2_ as the weak oxidant to regenerate the PC (Supplementary Fig. 27). The photoexcitation of eosin Y under blue (a wavelength of 455 nm) and green (525 nm) light yields similar conductivity values (Supplementary Fig. 28). Similar to our observations of PBTTT, the photocatalytic doping of P(g_4_2T-T) is a bulk process (Supplementary Fig. 29), resulting in a strong π–π stacking peak and an increase in the lamellar packing distance of the doped films, relative to the undoped films (Supplementary Figs. 30–32). Moreover, in contrast to other doping methods, photocatalytic doping yields P(g_4_2T-T) films with the highest crystallinity (Supplementary Figs. 33 and 34), accounting for the greater conductivity measured in this study. P3HT (which has an ionization potential of around 4.8 eV) was photocatalytically doped by Acr-Me^+^, Mes-Acr-Me^+^, Mes-Acr-Ph^+^ and perylene diimide, whereas the high-ionization-potential PTQ1 (ionization potential of around 5.3 eV) could be doped slightly by Acr-Me^+^, Mes-Acr-Me^+^ and Mes-Acr-Ph^+^ (Extended Data Fig. 9). The electrical-conductivity enhancement (with respect to the undoped state) for the six OSCs strongly depends on the energy difference between the ionization potential of the OSC and the EA^*^ of the PCs. As the energy difference decreases, the electrical-conductivity enhancement increases by several orders of magnitude (Fig. 3g).

## Photocatalytic n-doping and simultaneous p-doping and n-doping

**Fig. 4:**
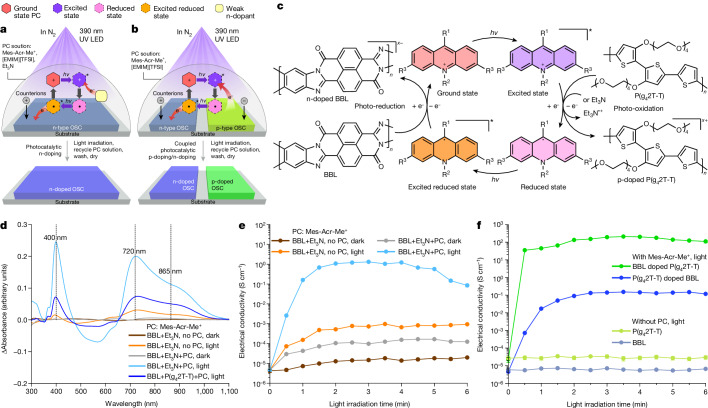
Photocatalytic n-doping and simultaneous photocatalytic p-doping and n-doping. **a**,**b**, Schematics of the photocatalytic n-doping process: BBL films are covered by the PC solution (0.01 M Mes-Acr-Me^+^ in 3:1 BuOAc:CH_3_CN) that also contains 0.1 M [EMIM][TFSI]. The weak n-dopant Et_3_N (0.1 M, **a**) or a physically separate p-type P(g_4_2T-T) film immersed in the PC solution (**b**) is used to regenerate the PC. After irradiation in N_2_, the PC solution is recovered, and the OSC films are washed with clean 3:1 BuOAc:CH_3_CN solvent and dried in N_2_. **c**, Proposed catalytic cycle for simultaneous photocatalytic p-doping and n-doping of OSCs. The photoactivated PC extracts electrons from the p-type P(g_4_2T-T) (or Et_3_N) and donates them to the n-type BBL. **d**, Differential absorption spectra of BBL films after photocatalytic n-doping by Mes-Acr-Me^+^ with Et_3_N or P(g_4_2T-T). **e**, Electrical conductivity of undoped and photocatalytically n-doped BBL films. **f**, Electrical conductivity of simultaneous photocatalytic p-doped P(g_4_2T-T) and n-doped BBL. Data in **e** and **f**: points, mean; error bars, s.d. (not visible); *n* = 10 independent samples. Source Data

Finally, we investigated the photocatalytic reduction (n-doping) and simultaneous photocatalytic p-doping and n-doping of OSCs (Fig. 4a,b). To achieve this, the PC was first photoactivated to generate an excited-state PC that can oxidize weak n-dopants. When the latter were oxidized, the PC was converted to its reduced form, which was subsequently photoactivated to obtain an excited reduced state capable of n-doping OSCs (Fig. 4c). In this process, the n-type OSC accepts an electron from the reduced or excited reduced state of the PC, enabling the regeneration of the ground-state PC.

To test this approach, we selected poly(benzimidazobenzophenanthroline) (BBL) as a prototypical n-type OSC with a high EA (4.30 eV, Supplementary Fig. 26), high electrical conductivity after doping (greater than 1 S cm^–1^)^30^ and high ion permeability^52^. Et_3_N was used as the weak n-dopant and [EMIM][TFSI] was used as the organic salt to stabilize the negative polarons on the n-doped BBL chains. Our results show that without light or PC, Et_3_N is only marginally able to n-dope BBL (Fig. 4d and Supplementary Fig. 35). However, in the presence of light and PC, Et_3_N considerably n-dopes BBL, resulting in strong negative polaron absorption peaks at 400 nm, 720 nm and 865 nm, similar to those observed in chemically^10,35^ and electrochemically^52^ n-doped BBL films. After photocatalytic n-doping, the electrical conductivity of BBL increases by more than five orders of magnitude, from less than 10^−5^ S cm^–1^ for undoped BBL to more than 1 S cm^−1^ after only 2 min of light irradiation (Fig. 4e). DFT computations again indicate that both the reduced and the excited reduced PC can reduce BBL, with the latter being more favoured (Supplementary Fig. 36).

**Extended Data Fig. 10:**
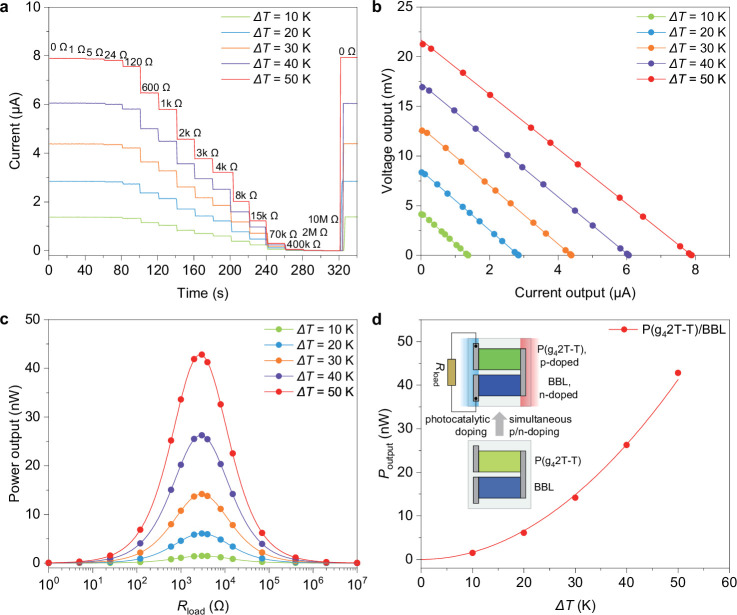
Use of simultaneous photocatalytic p/n-doping to build thermoelectric generators. **a**, Current of a planar thermoelectric module integrating one P(g_4_2T-T) p-leg (*W* = 40 mm, *L* = 2 mm, *d* = 20 μm) and one BBL n-leg (*W* = 20 mm, *L* = 2 mm, *d* = 10 μm) with silver contacts as a function of time for different load resistances (1-10^7^ Ω) and temperature gradients (∆*T* = 10-50 K). The devices show a stable, fast, and fully reversible response. The thermoelectric module was fabricated by drop-casting the p-type polymer P(g_4_2T-T) and n-type polymer BBL on a 25-μm polyethylene terephthalate (PET) substrate using a shadow mask. The P(g_4_2T-T) and BBL legs were doped by simultaneous photocatalytic p/n-doping using Mes-Acr-Me^+^ as the PC. A silver paste was used to print the contacts. The devices were tested in nitrogen. **b**, Voltage output of the thermoelectric module as a function of the load current (current output). **c**, Power output as a function of the resistance load recorded at different ∆*T*. **d**, Power output of the planar thermoelectric module as a function of ∆*T*.

By replacing Et_3_N with the low-ionization-potential polymer P(g_4_2T-T), we could couple the photocatalytic p-doping and n-doping of P(g_4_2T-T) and BBL, respectively. The semiconductor thin films, which were kept physically apart to prevent ground-state electron transfer^21^, were both immersed in the same Mes-Acr-Me^+^ solution and exposed to light to activate the PC (Fig. 4b). The Mes-Acr-Me^+^ serves as a redox shuttle, transferring electrons from P(g_4_2T-T) to BBL, as is evident from the distinctive polaronic features observed in the ultraviolet–visible spectra of both polymers (Fig. 4d and Supplementary Fig. 35). Photocatalytically p-doped P(g_4_2T-T) displays a typical conductivity of 200 S cm^−1^, whereas photocatalytically n-doped BBL shows a typical conductivity of 0.1 S cm^−1^ (Fig. 4e), with only [EMIM][TFSI] being consumed during the process to maintain charge neutrality. Importantly, p-doping or n-doping was observed only in the presence of Mes-Acr-Me^+^ (Fig. 4f). Although the inevitable alignment of the electrochemical potentials of the polymers (Supplementary Fig. 37) could thermodynamically limit the maximum doping level achievable through simultaneous photocatalytic p-doping or n-doping, this approach enables the concurrent oxidation and reduction of OSCs, which would otherwise be difficult to attain using conventional doping methods. The potential technological effect of this approach is demonstrated by fabricating a thermoelectric generator in which both p-type and n-type legs can be cast on a 25-μm plastic substrate from a solution in ambient conditions and simultaneously doped by Mes-Acr-Me^+^ to yield power outputs comparable to state-of-the-art thermoelectric modules in which the p-legs and n-legs are doped individually using conventional methods (Extended Data Fig. 10).

## Concluding remarks

We have reported a previously unpublished concept of photocatalytic doping for OSCs that offers a simple and efficient solution-based process at room temperature. The doping level can be easily controlled by adjusting the light irradiation dose. Compared with conventional doping methods that rely on highly reactive dopants that are consumed during the doping process, photocatalytic doping uses recyclable and air-stable PCs and consumes only TSFI-based salt and weak dopants, such as O_2_ (in air). This photocatalytic method is general and applicable to a wide range of OSCs, yielding p-doped, n-doped and simultaneously p-doped and n-doped OSCs with high electrical conductivity. Furthermore, it enables the direct insertion of redox-inert counterions into initially undoped OSC films without negatively affecting their microstructure. These results underscore the importance of photocatalytic doping for fundamental and applied research in organic electronics.

## Methods

### Materials

Acr-Me^+^ was purchased from TCI. Mes-Acr-Me^+^, Mes-Acr-Ph^+^, eosin Y, lithium bis(trifluoromethanesulfonyl)imide (LiTFSI), 1-ethyl-3-methylimidazolium bis(trifluoromethylsulfonyl)imide ([EMIM][TFSI]), diphenyl disulfide ((PhS)_2_), *n*-butyl acetate (BuOAc), acetonitrile (CH_3_CN), ether (Et_2_O), Et_3_N, 1,2-dichlorobenzene (ODCB), methanesulfonic acid (MSA) and dimethylformamide (DMF) were purchased from Sigma-Aldrich. Poly[2,3-bis(3-octyloxyphenyl)−5,8-quinoxalinediyl−2,5-thiophenediyl] (PTQ1) and poly(3-hexylthiophene-2,5-diyl) (P3HT) were purchased from Ossila. CH_3_CN was dried with a 3 Å molecular sieve before use. Acr-Me^+^, Mes-Acr-Me^+^ and Mes-Acr-Ph^+^ were recrystallized using a CH_3_CN/Et_2_O mixed solvent before use. All the other materials were used as received. Poly[2,5-bis(3-tetradecylthiophen-2-yl)thieno[3,2-b]thiophene] (PBTTT), alkylated perylene-diimide, glycolated poly-bithiophene-thiophene (P(g_4_2T-T)), poly-bithiophene-thienothiophene (P(g_4_2T-TT)) and poly-diketopyrrolopyrrole-thiophene (gDPP-g2T), as well as BBL, were synthesized using the procedure reported in refs. ^20,21,53–56^.

### Sample preparation

PTQ1, PBTTT, P3HT, gDPP-g2T, P(g_4_2T-TT) and P(g_4_2T-T) were dissolved in ODCB and spin-coated onto glass substrates to obtain the corresponding thin films. BBL was dissolved in MSA and spin-coated onto glass substrates, then immersed in water to remove residual MSA and dried with N_2_ flow to obtain BBL thin films. Unless otherwise stated, the typical PC solutions were as follows: (1) for p-doping with O_2_ as the p-dopant: BuOAc:CH_3_CN = 3:1 (volume ratio), [*C*_LiTFSI_] = 0.1 M, [*C*_PC_] = 0.01 M; (2) for p-doping with (PhS)_2_ as the p-dopant: BuOAc:CH_3_CN = 3:1, [*C*_LiTFSI_] = 0.1 M, [*C*_(PhS)2_] = 0.1 M, [*C*_PC_] = 0.01 M; (3) for n-doping with Et_3_N as the n-dopant: BuOAc:CH_3_CN = 3:1, [*C*_[EMEI][TFSI]_] = 0.1 M, [*C*_Et3N_] = 0.1 M, [*C*_PC_] = 0.01 M; (4) for simultaneous p-type and n-type doping: BuOAc:CH_3_CN = 3:1, [*C*_[EMEI][TFSI]_] = 0.1 M, [*C*_PC_] = 0.01 M. Typical solution preparation process: Acr-Me^+^, Mes-Acr-Me^+^, Mes-Acr-Ph^+^, perylene diimide or eosin Y were dissolved in dried CH_3_CN, then BuOAc was added to reach BuOAc:CH_3_CN = 3:1. For eosin Y, 1 vol% DMF was used to aid dissolution. The counterions LiTFSI (or [EMIM][TFSI]) were then added, followed by (PhS)_2_ or Et_3_N as in (2) and (3). For (1), the solution was saturated with air (by air bubbling) before use. For (2–4), all the solutions were processed in N_2_.

### Photocatalytic p-doping

OSC thin films were covered with PC solution and irradiated with 455 nm blue light. Unless otherwise stated, the light irradiation intensity was 50 mW cm^–2^. A heat sink was placed under the OSC films to avoid heating and to slow down solvent evaporation. In the case of O_2_, the photocatalytic doping process was performed in air. In the case of (PhS)_2_, the photocatalytic doping process was performed in N_2_. After light irradiation, the PC solution was recovered and the OSC films were washed with clean BuOAc:CH_3_CN = 3:1 mixed solvent and dried under N_2_ flow.

### Photocatalytic n-doping

The photocatalytic n-doping and simultaneous p-type and n-type doping processes were conducted in an N_2_-filled glovebox. The OSC thin films were covered with the PC solution and irradiated with both 455 nm blue light and 390 nm ultraviolet light. The light irradiation intensities for both blue light and ultraviolet light were 50 mW cm^−2^. A heat sink was placed under the OSC films to avoid heating and to slow down solvent evaporation. After light irradiation, the PC solution was recovered and the OSC thin films were washed with clean BuOAc:CH_3_CN = 3:1 mixed solvent and dried under N_2_ flow.

### Ultraviolet–visible–near-infrared absorption spectra

OSC thin films were prepared on glass substrates. Air-sensitive samples were placed in a sealed spectral cell filled with N_2_. Ultraviolet–visible–near-infrared absorption spectra of the films were measured using Perkin Elmer Lambda 900 with a resolution of 2 nm.

### UPS and XPS

OSC films were prepared on ITO-coated glass substrates. The XPS experiment used a Scienta ESCA 200 system with a base pressure of 2 × 10^–10^ mbar equipped with an SES 200 electron analyser, a monochromatic Al Ka X-ray source (1,486.6 eV) and a helium discharge lamp (21.22 eV) for XPS and UPS, respectively. All spectra were collected at normal emission and were calibrated by a sputter-cleaned Au film with the Fermi level at 0 eV and the Au(4 *f*) peak at 84.0 eV. The work function was extracted from the edge of the secondary electron cut-off while applying a −3 V bias on the sample, and the values were confirmed using Kelvin Probe (KP6500 McAllister Digital Kelvin Probe).

### Electrical characterization

Samples with high electrical conductivity (more than 1 S cm^–1^) were measured with a four-probe set-up (channel width/length was 10 mm/0.5 mm = 20). The results were validated using a smaller channel width/length ratio (0.5 mm/1 mm = 0.5; Supplementary Fig. 38). Samples with low electrical conductivity (less than 1 S cm^−1^) were measured using a two-probe setup with a channel width/length of 10 mm/0.5 mm = 20 or 200 μm/6 μm = 33.3. For channels with a width/length of 20, 5 nm of chromium as the adhesion layer and 50 nm of gold were thermally evaporated on cleaned glass substrates, the electrodes were patterned by photolithography and the OSC films were patterned by plastic tape. For channels with a width/length = 33.3, 5 nm of chromium as the adhesion layer and 50 nm of gold were thermally evaporated on cleaned glass substrates, the electrodes were patterned by photolithography and the OSC films were patterned using a double-layer of parylene C^57^. A layer of parylene C (1 μm) was deposited in the presence of 3-(trimethoxysilyl)propyl methacrylate (A-174 Silane) to increase adhesion. An antiadhesive layer of industrial surfactant (2% Micro-90) was spin-coated and a second layer of parylene C (sacrificial layer, 2 μm) was deposited. A 5-µm-thick AZ10XT520CP positive photoresist was then spin-coated on the second layer of parylene C. This protected the layers of parylene C from the following plasma reactive ion etching step (150 W, 500 standard cubic centimetres per minute O_2_, 100 standard cubic centimetres per minute CF_4_, 380 s). The contact pads and channel were defined by photolithography and AZ developer was applied to the photoresist. Plasma reactive ion etching removed the organic materials (photoresist and parylene C), exposing the channel area and the contact pads. The remaining surface was still covered with layers of parylene C. The OSC solution was spin-coated and the sacrificial layer of parylene C layer was then peeled off to remove the unwanted OSC film from outside the channel area. Electrical measurements used a Keithley 4200-SCS semiconductor characterization system. For n-doped OSCs, the conductivity measurements were conducted inside an N_2_-filled glovebox.

### Cyclic voltammetry

Cyclic voltammetry was measured on a Potentiostat BioLogic SP-200. A 0.1 M solution of n-Bu_4_NPF_6_ in anhydrous cBuOAc:CH_3_CN (3:1), bubbled with N_2_ for 1 min to remove the dissolved O_2_ before measuring, was used for Acr-Me^+^, Mes-Acr-Me^+^, Mes-Acr-Ph^+^, perylene diimide and a standard ferrocene sample. For eosin Y, 1 vol% DMF was added to the above electrolyte to aid dissolution. Glassy carbon was used as the working electrode and platinum mesh was used as the counter electrode, and Ag/AgCl (saturated) was used as the reference electrode. The scan rate was 50 mV s^–1^.

### Photoluminescence spectroscopy

The samples were excited at 3.1 eV (400 nm) using the second harmonic of a mode-locked Ti:sapphire laser (Mira 900, coherent) at a repetition rate of 76 MHz. The excitation beam was spatially limited by an iris and focused with a lens of focal length 150 mm. The fluence was adjusted using neutral density filters to 76 nJ cm^–2^. The excitation fluence per pulse was estimated assuming the diameter of the focused beam, *d*_focus_, is equal to 1.27 × *f*_lens_ *M*^2^/*D*, where *f*_lens_ is the focal length, *M* is the quality factor (assumed to be 1) and *D* is the incident diameter of the excitation beam. The spectra were taken in reflection geometry. The photoluminescence was collected using an achromatic doublet, where 425 nm and 435 nm long-pass filters were used to block the scattered laser light. Steady-state spectra were recorded with a Hamamatsu EM-CCD camera that was spectrally calibrated with a spectrograph equipped with a 50 lines per mm grating. Time-resolved traces were taken with a Hamamatsu streak camera working in single-sweep mode. A pulse picker was used to vary the repetition rate of the exciting pulses. The absolute photoluminescence quantum yields were measured using a Horiba Scientific Jobin Yvon spectrometer equipped with a Quanta-φ integrating sphere.

### Transient absorption spectroscopy

The differential transmission was measured by exciting the samples with an ultrafast laser source (100-fs laser pulses at 390 nm) obtained from a Ti:sapphire regenerative amplifier (Coherent Libra) with a repetition rate of 1 kHz and an optical parametric amplifier (Topas 800, Light Conversion). The pump pulses had a fluency of the order of 100 µJ cm^–2^ adjusted to have the best signal-to-noise ratio while avoiding sample degradation. ∆*T*/*T* measurements were performed by probing the samples with fs-supercontinuum white pulses (with a spectral range of 400–800 nm) at different time delays with respect to the excitation (pump) pulses, in a pump-probe configuration in which a transmitted beam and a reference beam were acquired with two CMOS grating spectrometers with 1 nm of spectral resolution (Ultrafast Systems Helios). The overall temporal resolution was always of the same order of magnitude as the excitation pulses (120 fs, full width at half maximum).

### GIWAXS

OSC thin films were prepared on Si/SiO_2_ substrates with an SiO_2_ layer thickness of 200 nm. GIWAXS experiments were performed at Beamline 9A U-SAXS at the Pohang Accelerator Laboratory in South Korea. The X-ray energy was 11.09 eV and the incidence angle was 0.12°. The samples were measured in a vacuum and the total exposure time was 10 s. The scattered X-rays were recorded by a CCD detector located 222.1186 mm from the sample. All samples for GIWAXS measurements had a similar thickness of around 60 nm.

### DFT

Calculations were done using the M06 hybrid meta-GGA functional^58^. The standard all-electron 6-311 G** basis was used for all atoms^59^. Molecular geometry optimization of stationary points was carried out without symmetry constraints and used analytical gradient techniques. Frequency analysis was done to obtain thermochemical information about the reaction pathways at 298 K using the harmonic approximation. A single-point calculation of the optimized structures was carried out by adopting the aug-cc-pVTZ basis set^60^. The diffuse function was necessary because of the presence of anionic species along the reaction mechanism. Finally, solvent effects were modelled at the aug-cc-pVTZ level within the polarized continuum model^61^ and the SMD variation^62^. The polymers were modelled by a simplified dimer structure. All calculations were performed using the G16 (ref. ^63^) code on Linux cluster systems.

## Online content

Any methods, additional references, Nature Portfolio reporting summaries, source data, extended data, supplementary information, acknowledgements, peer review information; details of author contributions and competing interests; and statements of data and code availability are available at 10.1038/s41586-024-07400-5.

### Supplementary information

Supplementary Information This file contains Supplementary Figs. 1-38, Supplementary Table 1 and Supplementary References.

Supplementary Video 1 This video shows the photocatalytic doping of a PBTTT thin film by Acr-Me^+^ in air.

Supplementary Video 2 This video shows the regeneration of Mes-Acr-Me^+^ and Mes-Acr-Ph^+^ in air.

### Source data

Source Data Fig. 2

Source Data Fig. 3

Source Data Fig. 4

## Data Availability

Data supporting the findings of this study are available in the paper and the Supplementary Information files. Source data are provided with this paper.
