# Supplementary material for: Photocatalytic doping of organic semiconductors
Source: Nature. 2024 May 15;630(8015):96–101. doi: 10.1038/s41586-024-07400-5 (PMC11153156; doi:10.1038/s41586-024-07400-5)
Supplement: Supplementary file 1 — This file contains Supplementary Figs. 1-38, Supplementary Table 1 and Supplementary References. [file 41586_2024_7400_MOESM1_ESM.pdf]

nature portfolio

https://doi.org/10.1038/s41586-024-07400-5

Supplementary information

Photocatalytic doping of organic semiconductors

In the format provided by the authors and unedited

Supplementary Information

Photocatalytic doping of organic semiconductors

Wenlong Jin[1], Chi-Yuan Yang[1,2], Riccardo Pau[3,4], Qingqing Wang[1,2], Eelco K. Tekelenburg[3], Han-Yan Wu[1], Ziang Wu[5], Sang Young Jeong[5], Federico Pitzalis[4], Tiefeng Liu[1,6], Qiao He[7], Qifan Li[1], Jun-Da Huang[1], Renee Kroon[1], Martin Heeney[7], Han Young Woo[5], Andrea Mura[4], Alessandro Motta[8], Antonio Facchetti[9], Mats Fahlman[1], Maria Antonietta Loi[3], Simone Fabiano[1,2,6]**

[1]Laboratory of Organic Electronics, Department of Science and Technology, Linköping University, Norrköping, Sweden
[2]n-Ink AB, Bredgatan 33, 60221 Norrköping, Sweden
[3]Zernike Institute for Advanced Materials, University of Groningen, Nijenborgh 4, 9747 AG, Groningen, The Netherlands
[4]Dipartimento di Fisica, Università degli Studi di Cagliari, Monserrato, I-09042 Italy
[5]Department of Chemistry, College of Science, Korea University, 145 Anam-ro, Seongbuk-gu, Seoul 136-713, Republic of Korea
[6]Wallenberg Initiative Materials Science for Sustainability, Department of Science and Technology, Linköping University, SE-60174 Norrköping, Sweden.
[7]Department of Chemistry and Centre for Processable Electronics, Imperial College London, London, UK
[8]Dipartimento di Chimica, Università di Roma "La Sapienza", p.le A. Moro 5, Rome I-00185, Italy
[9]School of Materials Science and Engineering, Georgia Institute of Technology, Atlanta, Georgia 30332, USA

*e-mail: chi-yuan.yang@liu.se, simone.fabiano@liu.se

Table of Contents:

Supplementary Figs. 1-38

Supplementary Tables 1

Supplementary References

Supplementary Figs. 1-38

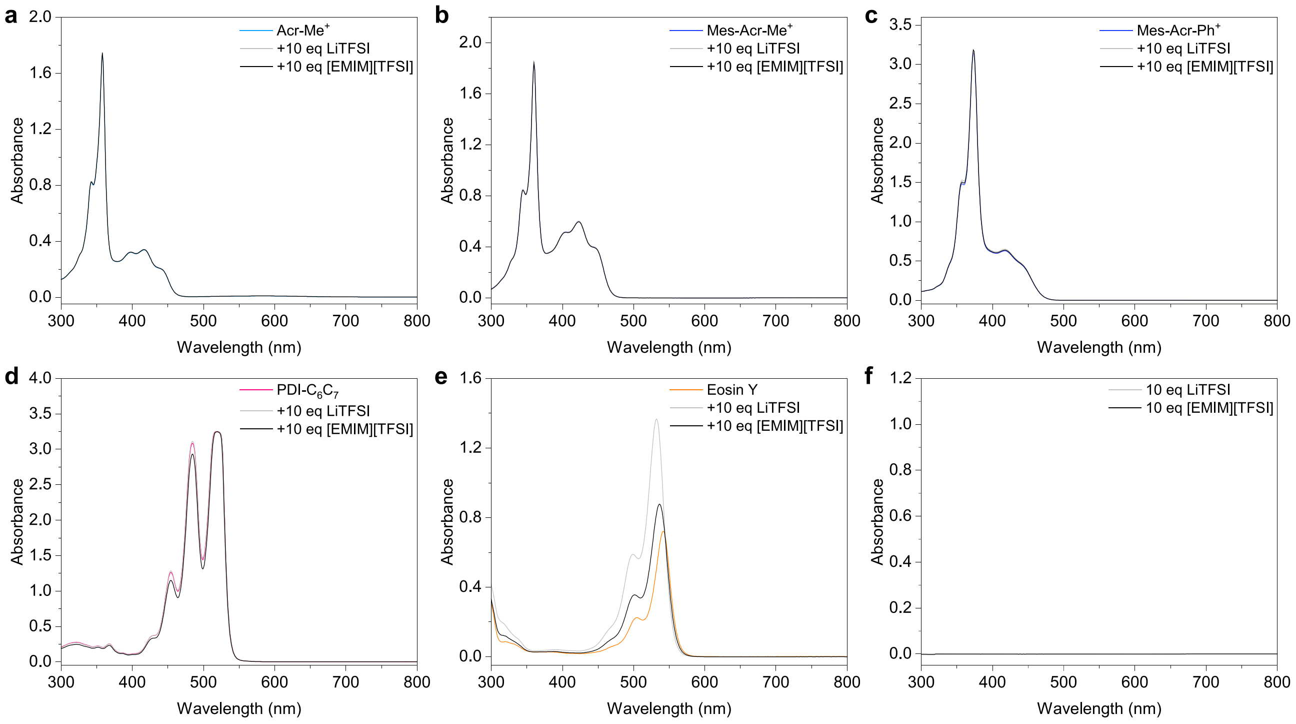

Supplementary Fig. 1 | Ionic metathesis. a-e, Absorption spectra of the photocatalyst Acr-Me$^+$ (**a**), Mes-Acr-Me$^+$ (**b**), Mes-Acr-Ph$^+$ (**c**), PDI-C_6C_7 (**d**), and Eosin Y (**e**) in BuOAc:CH_3CN (3:1) solution with and without 10 eq of LiTFSI or [EMIM][TFSI]. **f**, Absorption spectra of pure LiTFSI or [EMIM][TFSI] in BuOAc:CH_3CN (3:1) solution. Mixing the photocatalyst with the electrolyte does not change the aggregate state of most photocatalysts, with the exception of Eosin Y.

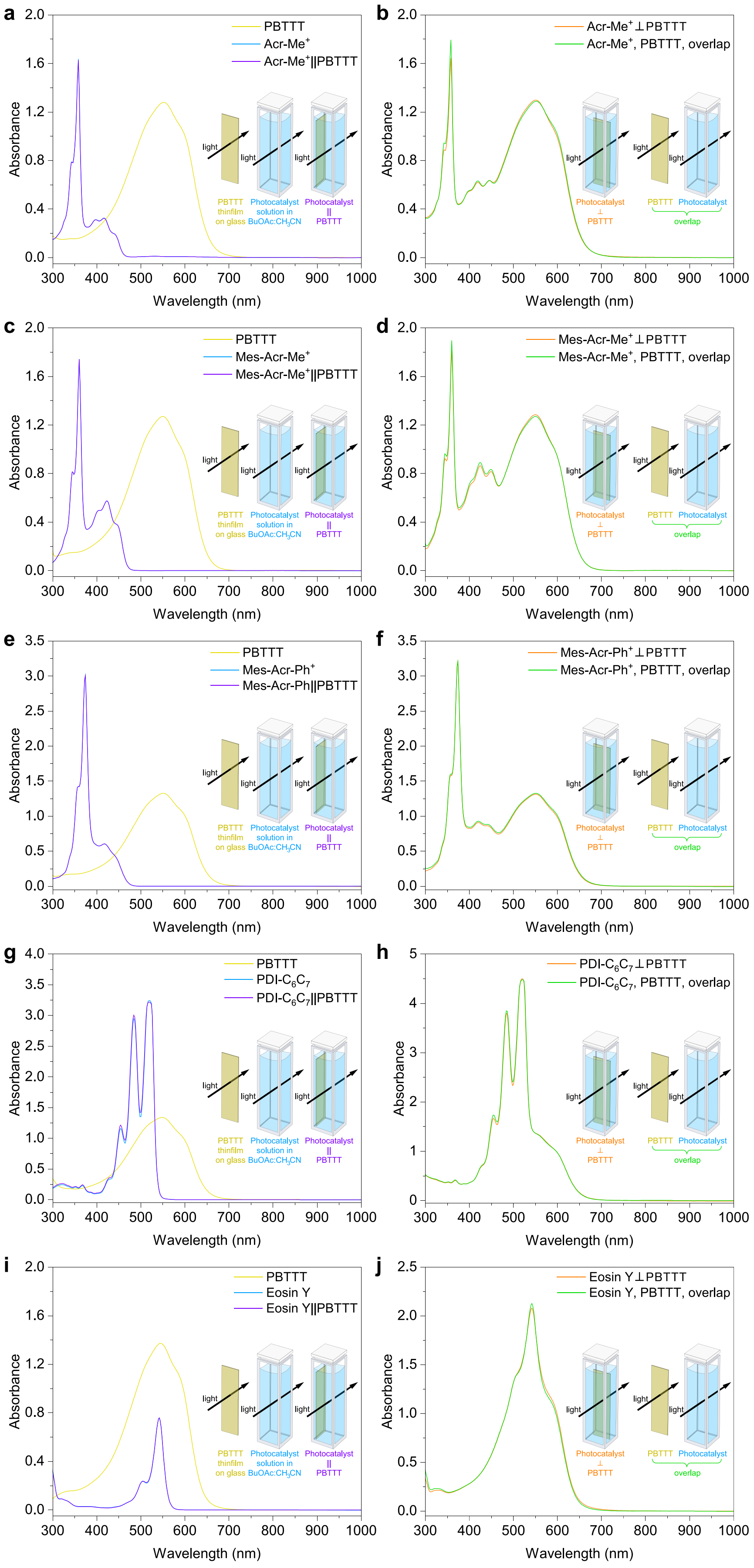

Supplementary Fig. 2 | Photocatalyst-semiconductor interaction. a-j, Absorption spectra of PBTTT thin films, the photocatalysts in solution (BuOAc:CH_3CN 3:1) for Acr-Me^+ (**a-b**), Mes-Acr-Me^+ (**c-d**), Mes-Acr-Ph^+ (**e-f**), PDI-C_6C_7 (**g-h**), and Eosin Y (**i-j**). Yellow curve: PBTTT thin films;

Blue curve: photocatalyst solution; Purple curve: PBTTT film and photocatalyst solution with PBTTT film not in the light pathway; Orange curve: PBTTT film and photocatalyst solution with PBTTT film in the light pathway; Green curve: sum of PBTTT thin film and photocatalyst solution absorptions. No changes in the absorption spectra are visible, indicating no significant interaction at the ground state between the photocatalysts and the organic semiconductor.

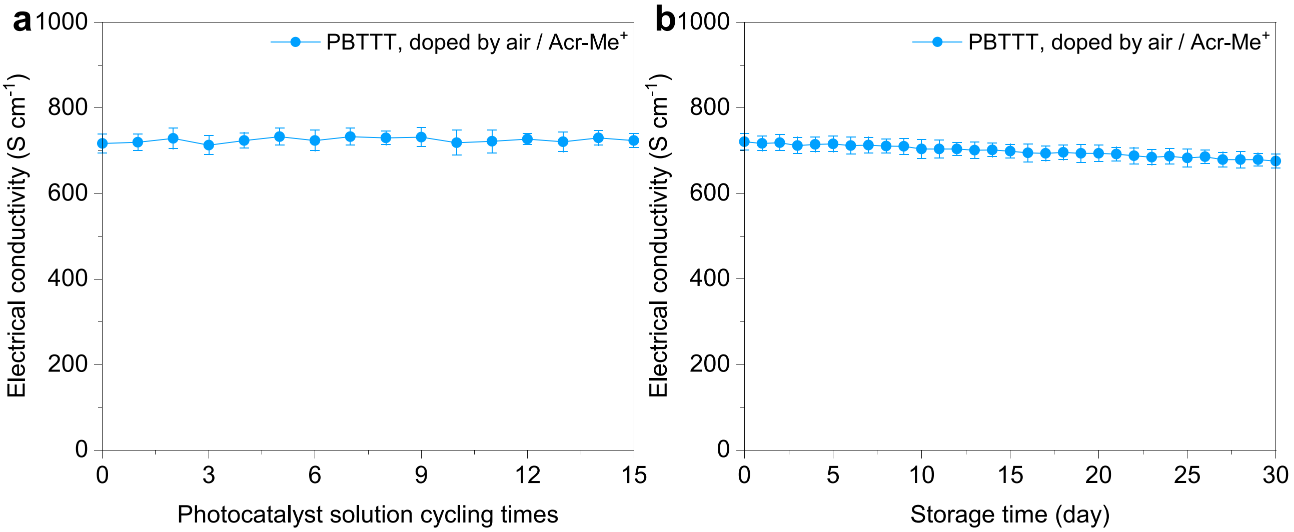

Supplementary Fig. 3 | Photocatalyst recyclability and stability. a, Recovery of the photocatalyst solution. No decrease in photocatalytic activity was observed following 15 cycles using the recovered catalyst solution. **b**, Stability of photocatalytically p-doped PBTTT (by Acr-Me^{+}/air), demonstrating that >95% of the electrical conductivity remains after storage (in dark and under nitrogen to prevent oxidation by air) for 30 days.

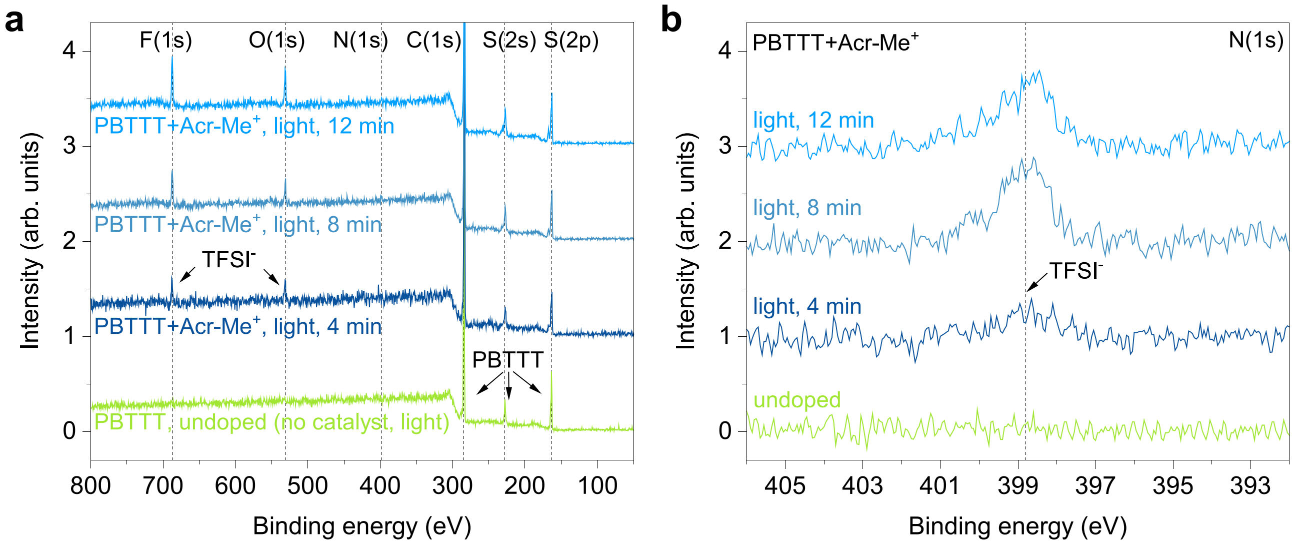

Supplementary Fig. 4 | X-ray photoelectron spectroscopy (XPS) analysis. a, XPS analysis of undoped PBTTT and doped PBTTT (photocatalyzed by Acr-Me^{+}) thin films under different 455 nm light irradiation time. **b,** N(1s) XPS spectra undoped PBTTT and doped PBTTT thin films under different light irradiation time. XPS spectra shows increased TFSI^{-} intensity in PBTTT films after light irradiation, indicating effective photocatalytic doping.

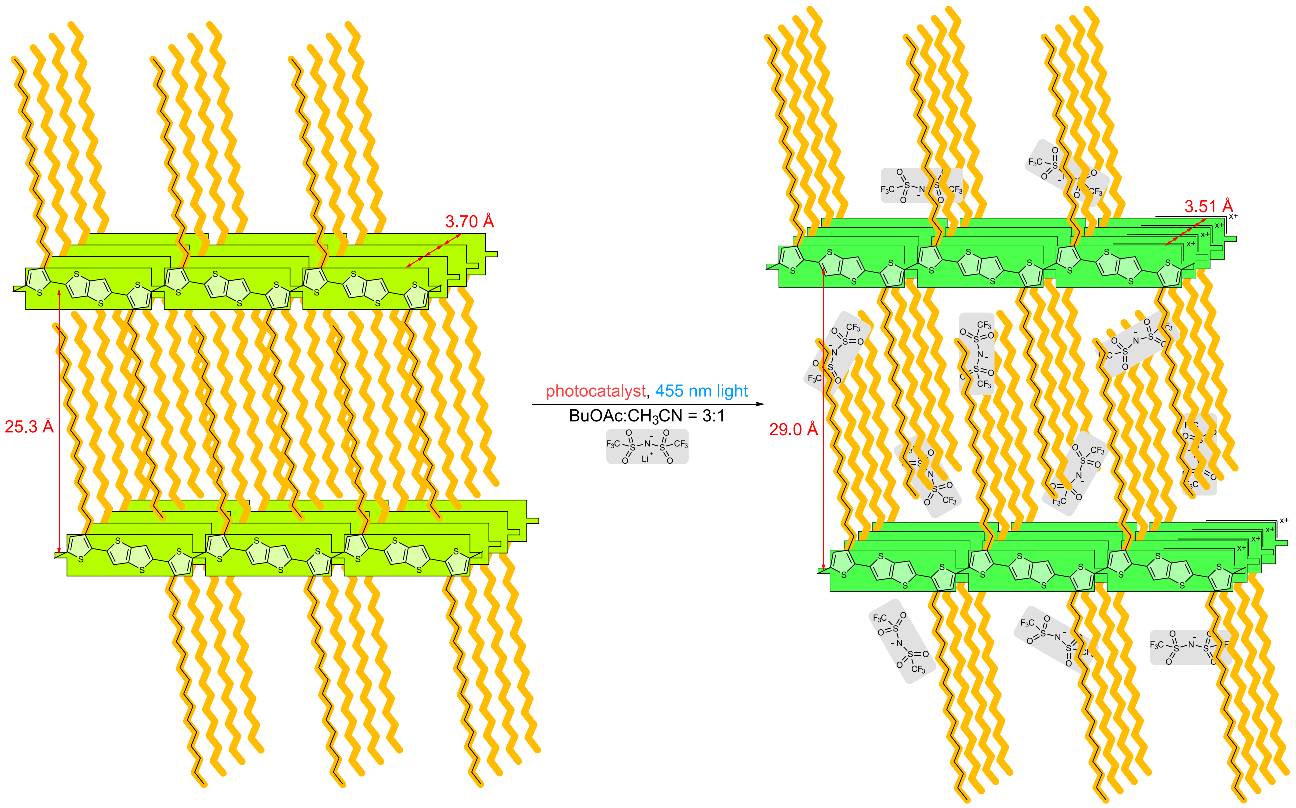

Supplementary Fig. 5 | Molecular packing in photocatalytic doping. Schematic diagram of undoped and photocatalytically doped PBTTT thin films. Photocatalytically doped PBTTT shows longer lamellar packing distance, shorterπ-π stacking distance, and more regular π-π stacking than undoped PBTTT, which implies that the counterions are located in the side chain packing region of doped PBTTT.

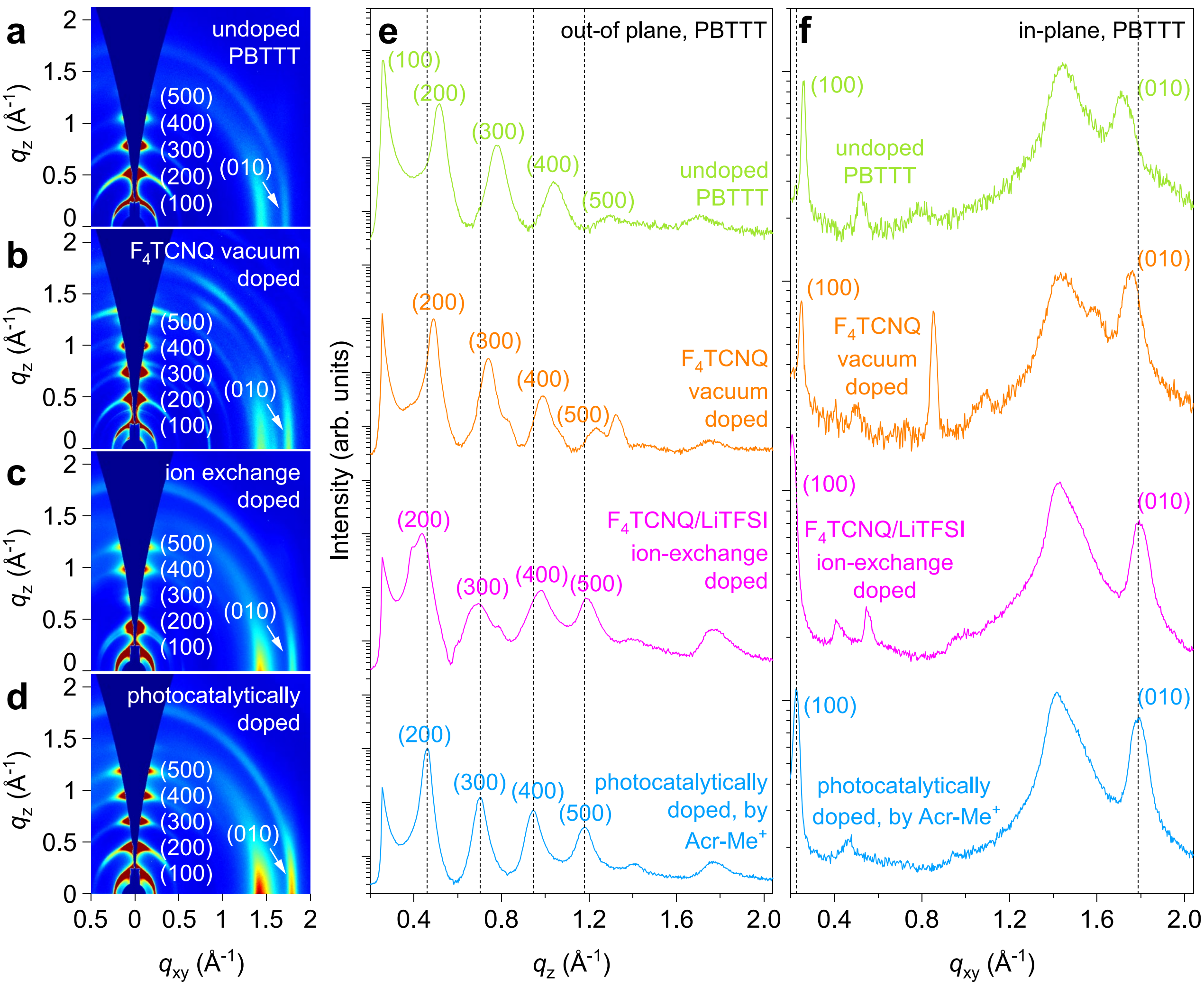

Supplementary Fig. 6 | GIWAXS patterns and line cuts of photocatalytically p-doped PBTTT and comparison with other doping methods. a-d, 2D GIWAXS patterns of undoped PBTTT (**a**), F_4TCNQ vacuum doped PBTTT (**b**), F_4TCNQ/LiTFSI ion exchange doped PBTTT (**c**) and Acr-Me^+/air photocatalyzed doped PBTTT (**d**) thin films. **e-f,** Out-of-plane (**e**) and in-plane (**f**) GIWAXS line cuts of undoped and doped PBTTT thin films.

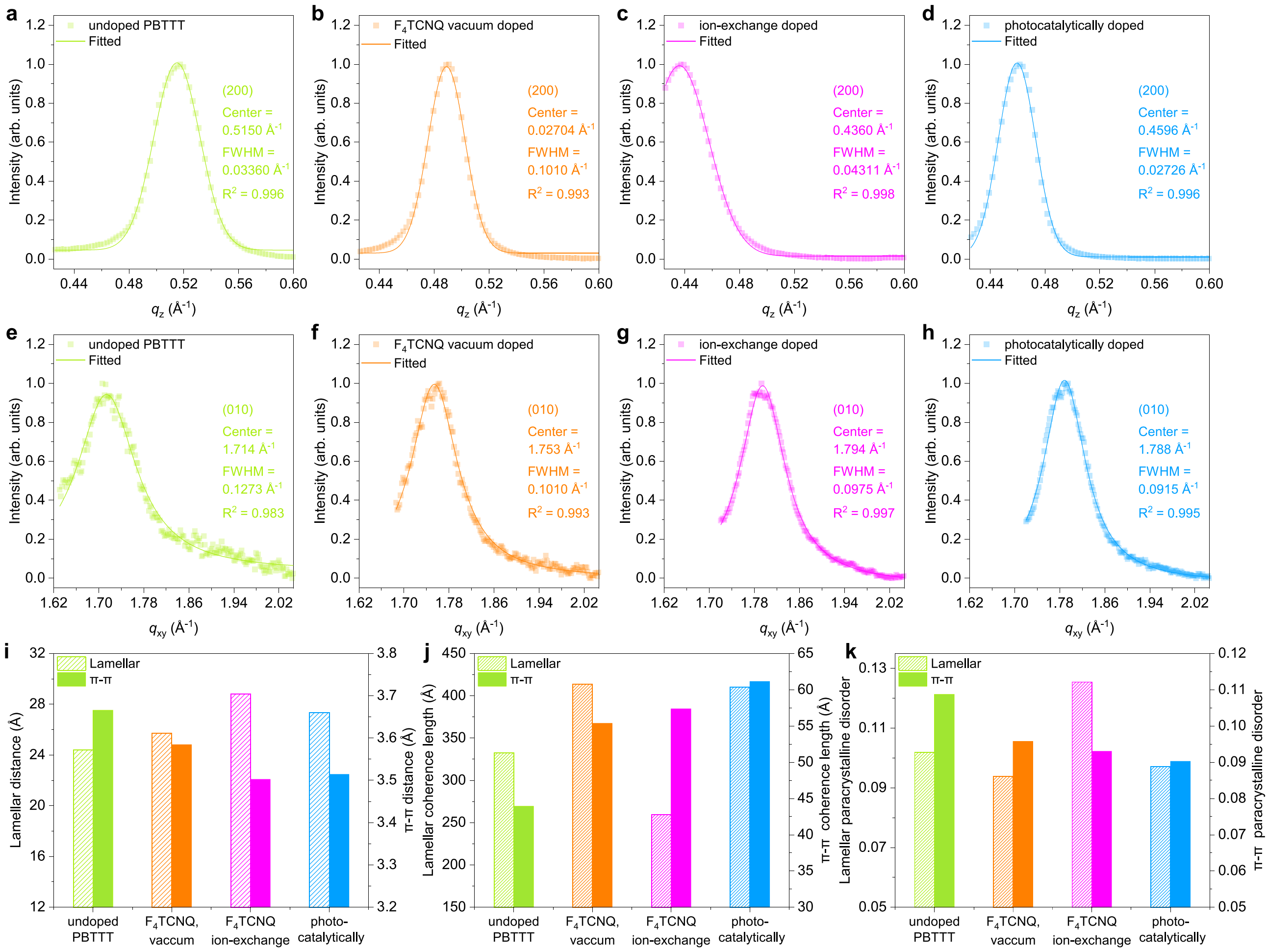

Supplementary Fig. 7 | Crystallinity of PBTTT for the different doping methods. a-d, Lamellar packing diffraction peak analysis of undoped PBTTT (**a**), F_4TCNQ vacuum doped PBTTT (**b**), F_4TCNQ/LiTFSI anion exchanging doped PBTTT (**c**) and Acr-Me^+/air photocatalyzed doped PBTTT (**d**) thin films. **e-h,** π-π stacking diffraction peak analysis of undoped PBTTT (**e**), F_4TCNQ vacuum doped PBTTT (**f**), F_4TCNQ/LiTFSI ion exchange doped PBTTT (**g**) and Acr-Me^+/air photocatalyzed doped PBTTT (**h**) thin films. **i-k,** Packing distances (**i**), coherence length (**j**), and paracrystalline disorder (**k**) of lamellar packing and π-π stacking of PBTTT films. Compared to the other doping methods, the photocatalytic doping method yields doped PBTTT films with the highest crystallinity. Note that the values in **i-k,** extracted from the GIWAXS line cuts of Supplementary Fig. 6, differ slightly from those reported in Extended Data Fig. 3, as these measurements were conducted during a different beamtime session.

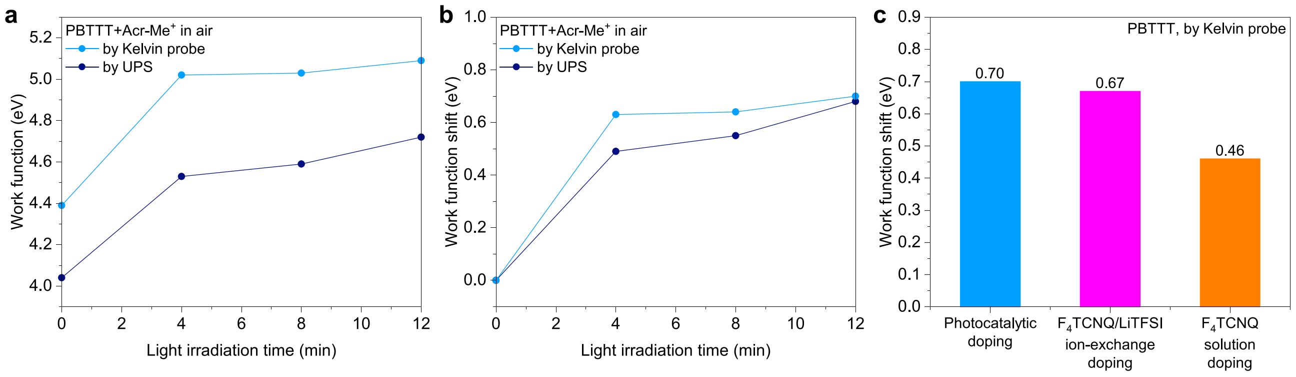

Supplementary Fig. 8 | Work function shift in p-doped PBTTT films. a, Work function of photocatalytically p-doped PBTTT by Acr-Me$^+$ in air, measured by UPS and Kelvin Probe as a function of the irradiation time. **b**, Relative work function shift. **c**, Comparison of the work function shift for different doping methods.

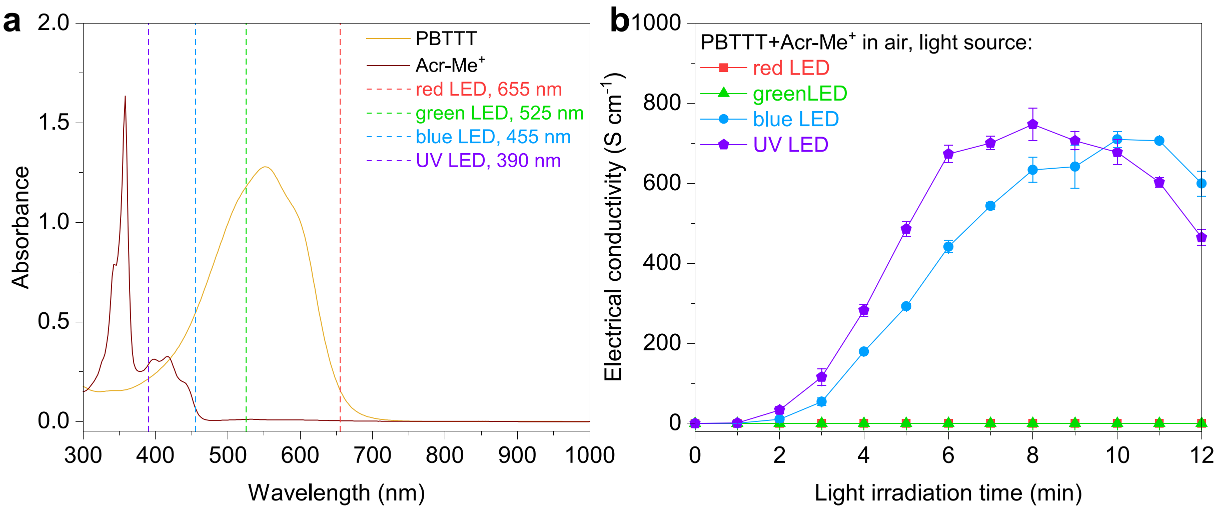

Supplementary Fig. 9 | Effect of excitation wavelength. a, Absorption spectra of Acr-Me$^+$ and PBTTT. **b**, Electrical conductivity of PBTTT photocatalytically doped with Acr-Me$^+$ in air under UV (390 nm), blue (455 nm), green (525 nm), and red (655 nm) light irradiation. Note that while PBTTT can be excited by all light sources, Acr-Me$^+$ can be only excited by UV and blue lights.

Spectroscopic characterization

Transient absorption spectra of undoped PBTTT thin films reveal the presence of photobleaching peaks in the range of 550-575 nm, along with a photoinduced absorption peak at 638 nm (Supplementary Fig. 10). By monitoring the decay of these features associated with excited state absorption/bleaching, we observed a relatively short excited state lifetime of 46-60 ps for the undoped PBTTT (Supplementary Fig. 10). Upon photocatalytic doping with Acr-Me$^+$, we observed an even shorter excited state lifetime (19-24 ps) for doped PBTTT (Supplementary Fig. 11). In contrast, Acr-Me$^+$ exhibited an excited state lifetime of 6-9 ns, which is 100× longer than that of PBTTT (see Supplementary Fig. 12). This significant difference in excited state lifetimes is also reflected in their fluorescence lifetimes (see Supplementary Figs. 13-16), that for Acr-Me$^+$ is much longer (18 ns) than that of PBTTT (0.9 ns). Note that all photocatalysts show systematically longer fluorescence lifetimes than the organic semiconductors (Supplementary Fig. 17).

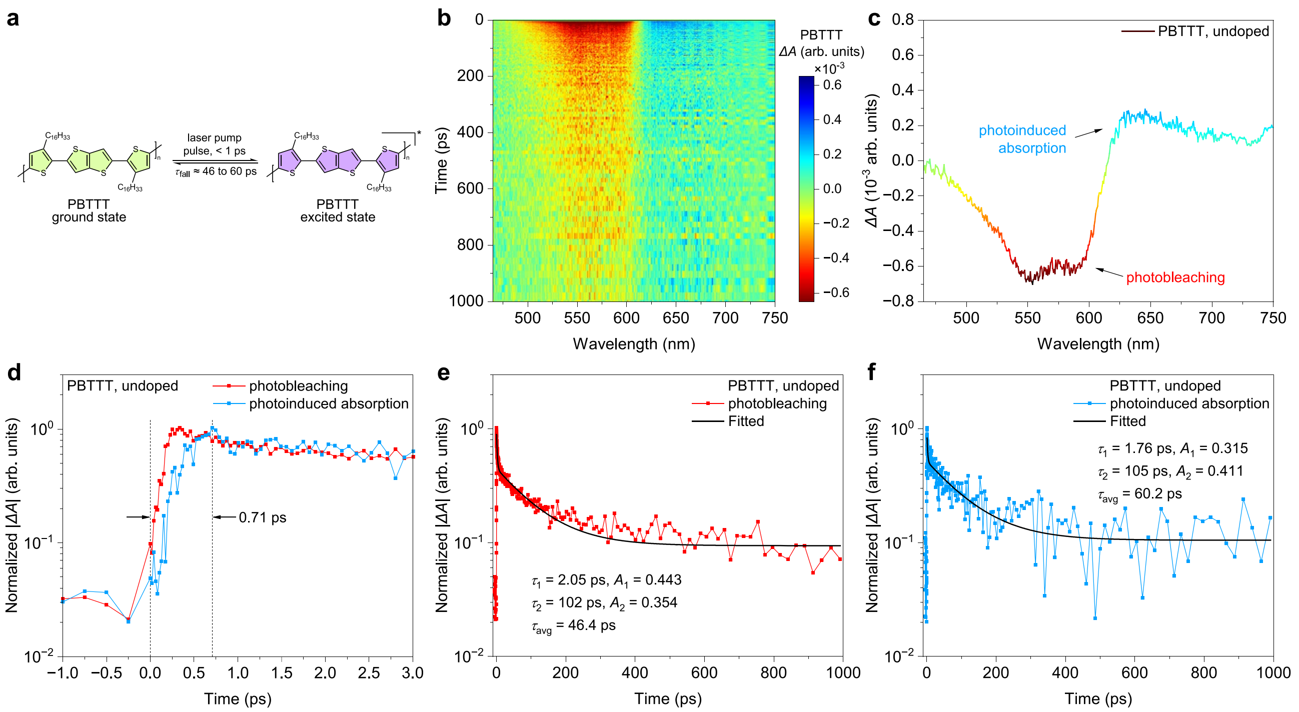

Supplementary Fig. 10 | Transient absorption spectra (TAS) of undoped PBTTT. a, Schematic diagram of the ground-to-excited state transition of undoped PBTTT under 390 nm laser pump pulse. **b,** False color representation of the transient absorption spectra of undoped PBTTT thin film as a function of delay time and detection wavelength. **c**, Transient absorption spectra of undoped PBTTT thin film showing photobleaching (peak center 638 nm) and photoinduced absorption (peak center 553-574 nm). **d-f,** Time-dependent photobleaching and photoinduced absorption of the undoped PBTTT thin film, showing immediate raising (< 1 ps, limited by instrumental time resolution, **d**), followed by a decay dynamic with a short excited lifetime of 46-60 ps (**e-f**).

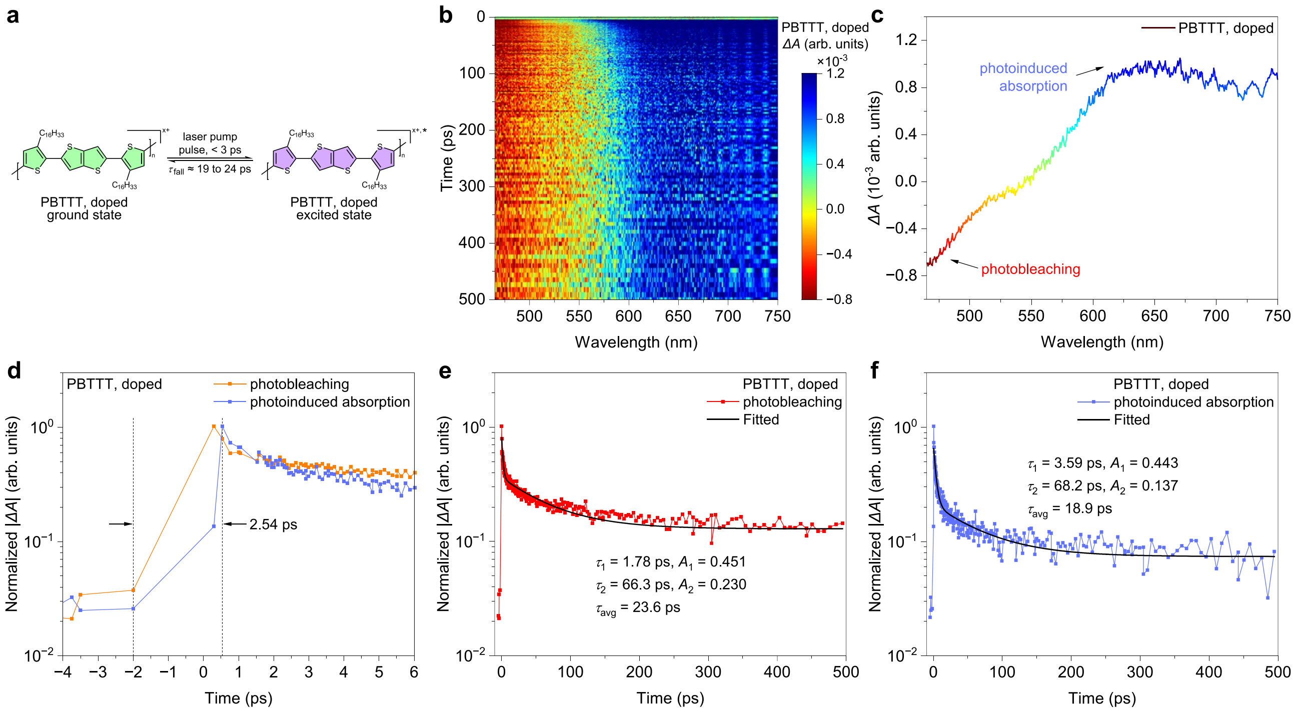

Supplementary Fig. 11 | TAS of doped PBTTT. **a**, Schematic diagram of the ground-to-excited state transition of photocatalytically doped PBTTT by Acr-Me$^+$/air, under 390 nm laser pump pulse. **b**, False color representation of the transient absorption spectra of doped PBTTT thin film as a function of delay time and detection wavelength. **c**, Transient absorption spectra of doped PBTTT thin film showing the photobleaching (465-544 nm) and photoinduced absorption (peak center 647 nm). **d-f**, Time-dependent photobleaching and photoinduced absorption of doped PBTTT thin film, showing immediate raising (< 3 ps, limited by instrumental time resolution, **d**), followed by a decay dynamic with a short excited lifetime of 19-24 ps (**e-f**).

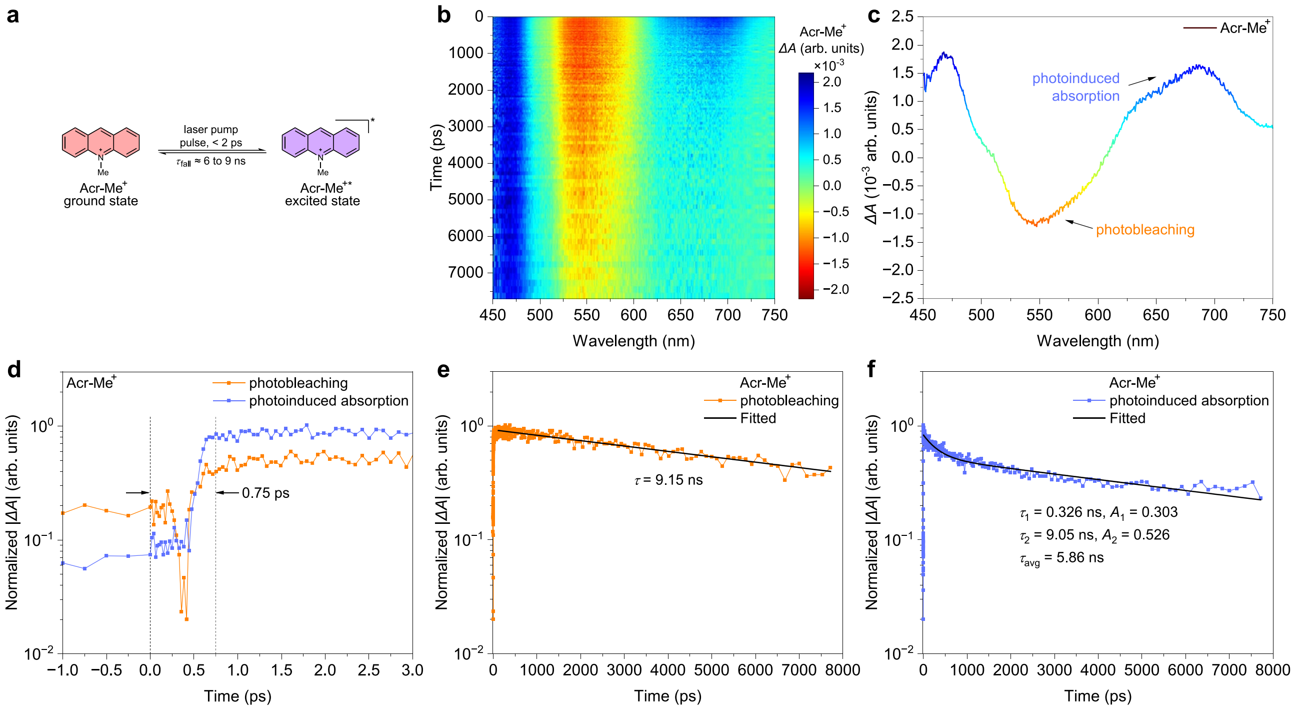

Supplementary Fig. 12 | TAS of Acr-Me$^+$. a, Schematic diagram of the ground-to-excited state transition of Acr-Me$^+$ under 390 nm laser pump pulse. **b**, False color representation of the transient absorption spectra of Acr-Me$^+$ solution (0.01 M, with 0.1 M of LiTFSI, in BuOAc:CH_3CN = 3:1) as a function of delay time and detection wavelength. **c**, Transient absorption spectra of Acr-Me$^+$ showing the significant photobleaching (peak center 546 nm) and photoinduced absorption (peak center 686 nm). **d-f**, Time-dependent photobleaching and photoinduced absorption of Acr-Me$^+$, showing immediate raising (< 1 ps, limited by instrumental time resolution, **d**), followed by a decay dynamic with a long excited lifetime of 6-9 ns (**e-f**). The excited lifetime of photocatalyst Acr-Me$^+$ is around 100 times longer than that of semiconductor PBTTT thin film.

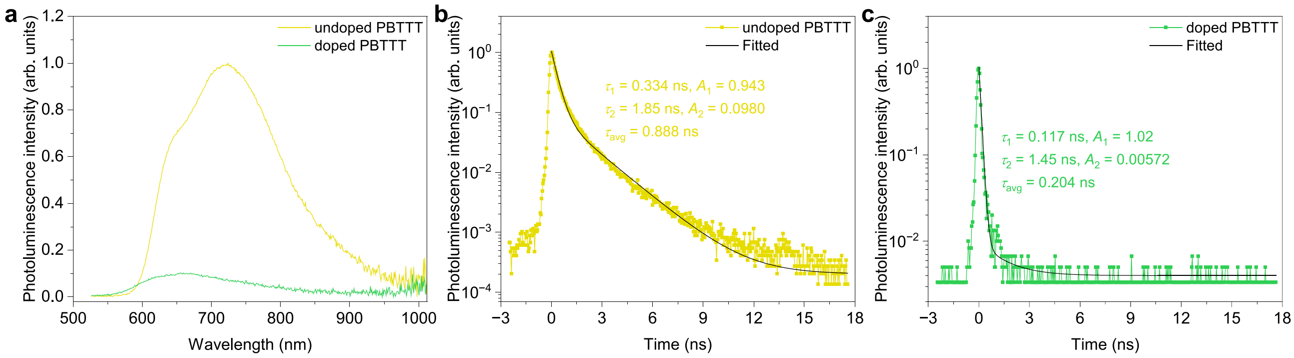

Supplementary Fig. 13 | Photoluminescence spectra of PBTTT. a, Photoluminescence spectra of undoped and doped (photocatalyzed by Acr-Me$^+$/air) PBTTT thin films. **b-c**, Time-resolved photoluminescence spectra of undoped (**b**) and doped (**c**) PBTTT, showing short fluorescence lifetime of 0.9 ns and 0.2 ns (for undoped and doped PBTTT, limited by instrumental time resolution). The excitation wavelength is 400 nm.

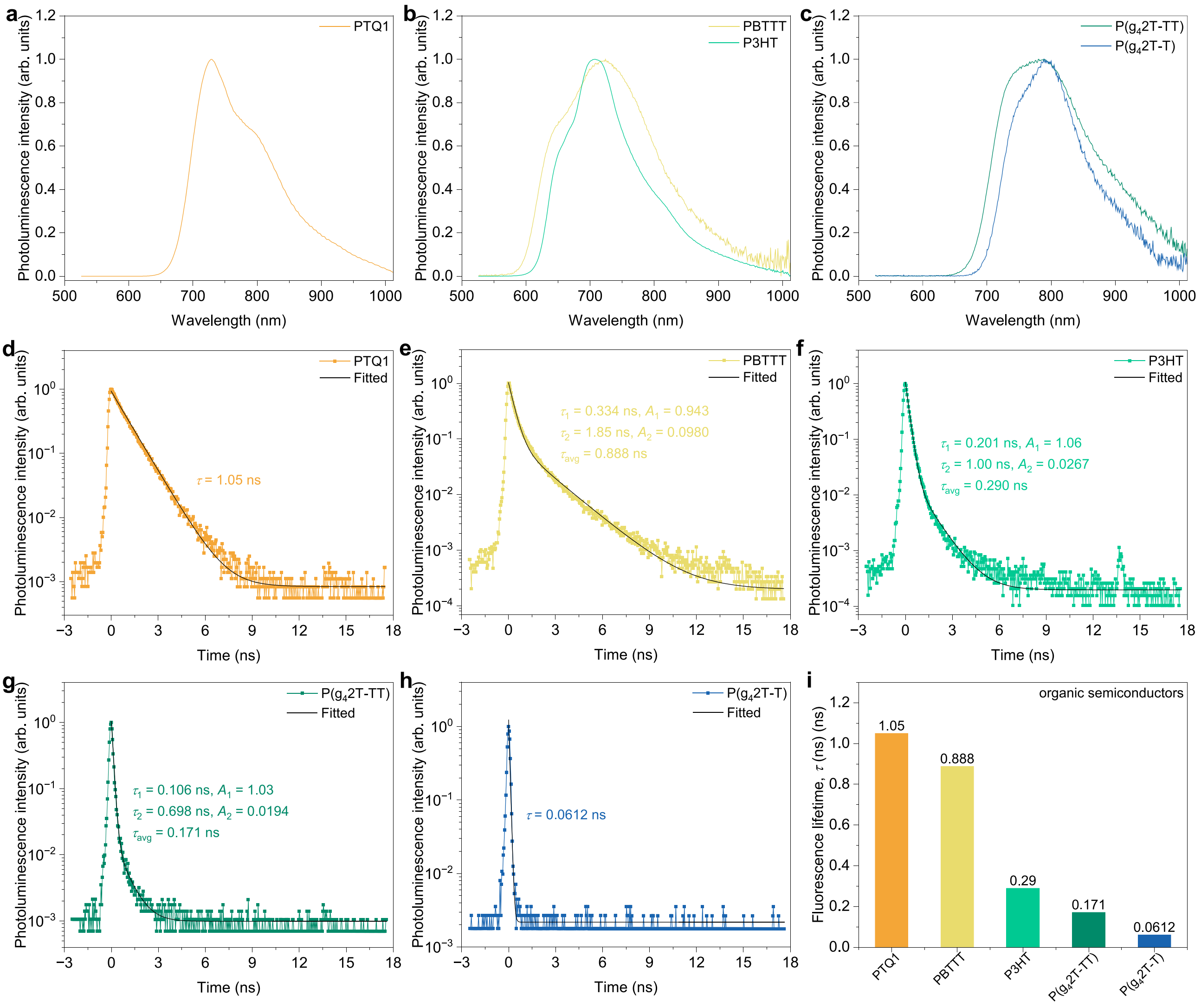

Supplementary Fig. 14 | Photoluminescence spectra of organic semiconductors. a-c, Photoluminescence spectra of undoped PTQ1 (**a**), PBTTT and P3HT (**b**), and P(g$_4$2T-TT) and P(g$_4$2T-TT) (**c**) thin films. **d-f**, Time-resolved photoluminescence spectra of PTQ1 (**d**), PBTTT (**e**), P3HT (**f**), P(g$_4$2T-TT) (**g**), and P(g$_4$2T-T) (**h**) thin films. **i**, Fluorescence lifetime of the above organic semiconductors. The fluorescence lifetime of P(g$_4$2T-TT) and P(g$_4$2T-T) are limited by instrumental time resolution. The excitation wavelength is 400 nm.

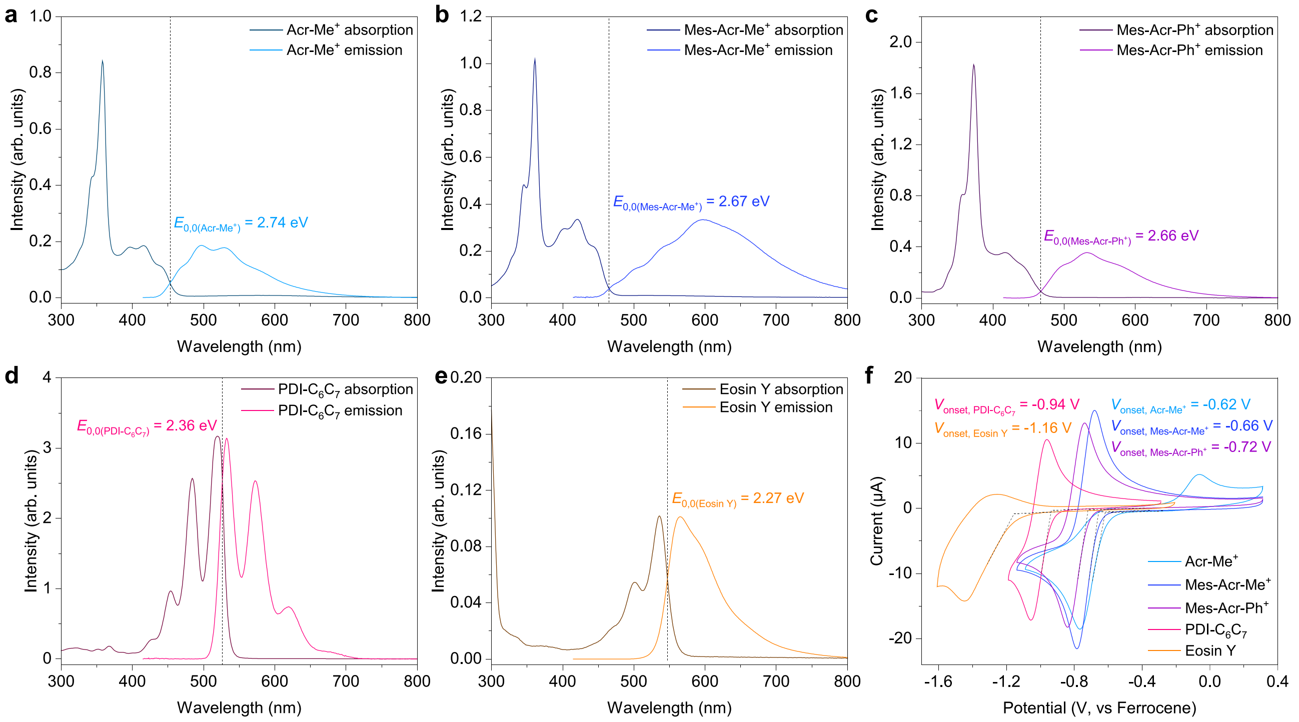

Supplementary Fig. 15 | Photoluminescence spectra and electron affinity of photocatalyst. a-e, Absorption and photoluminescence spectra of Acr-Me$^+$ (**a**), Mes-Acr-Me$^+$ (**b**), Mes-Acr-Ph$^+$ (**c**), PDI-C_6C_7 (**d**), and Eosin Y (**e**) in diluted solution (BuOAc:CH_3CN = 3:1). The photoluminescence spectra are excited at 400 nm. **f,** Cyclic voltammetry scan of the above photocatalysts in BuOAc:CH_3CN mix solution. Scan speed is 50 mV s^{-1}. The ground state *EA* of photocatalyst was calculated from $EA_{\text{ground state}} = (4.80 + V_{\text{onset, reduction, vs Ferrocene}}/\text{V})$ eV. The excited *EA* of photocatalyst is calculated from $EA_{\text{excited state}} = (4.80 + V_{\text{onset, reduction, vs Ferrocene}}/\text{V} + E_{0,0})$ eV. $E_{0,0}$ is the adiabatic S_1 state energy level, which is approximated with the crossing point of absorption and fluorescence emission spectra (for the singlet excited state) after normalization[1].

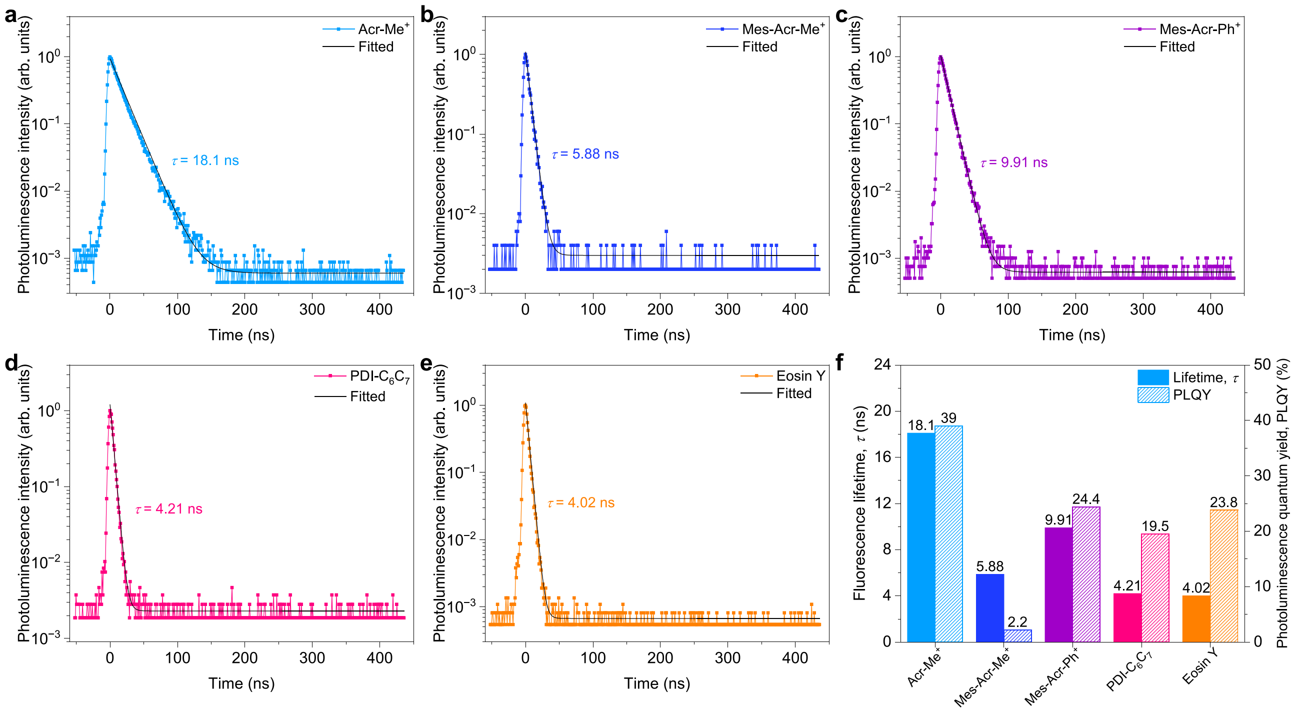

Supplementary Fig. 16 | Fluorescence lifetime. a-e, Time-resolved photoluminescence spectra of Acr-Me^+ (**a**), Mes-Acr-Me^+ (**b**), Mes-Acr-Ph^+ (**c**), PDI-C_6C_7 (**d**), and Eosin Y (**e**) in solution (BuOAc:CH_3CN = 3:1) with LiTFSI (0.1 mol L^{-1}). **f,** Fluorescence lifetime and photoluminescence quantum yield (PLQY)of the above photocatalysts. The excitation wavelength is 400 nm.

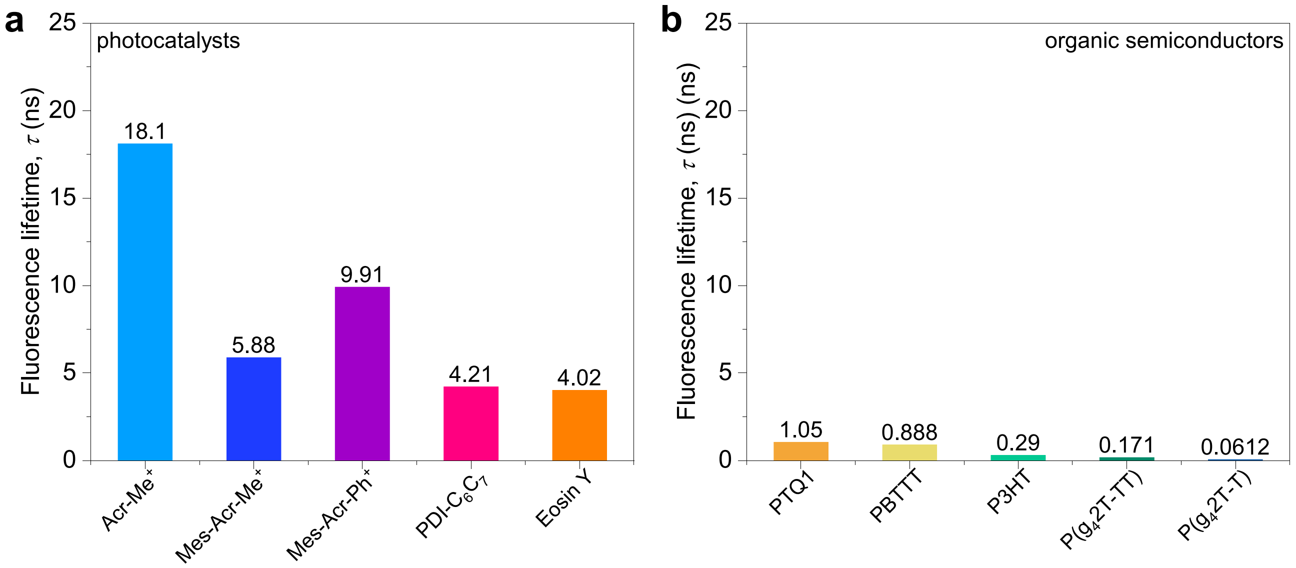

Supplementary Fig. 17 | Fluorescence lifetime. a-b, Fluorescence lifetime of photocatalysts (**a**) and organic semiconductors (**b**). The excitation wavelength is 400 nm. The fluorescence lifetime of the photocatalysts is much longer than that of organic semiconductors.

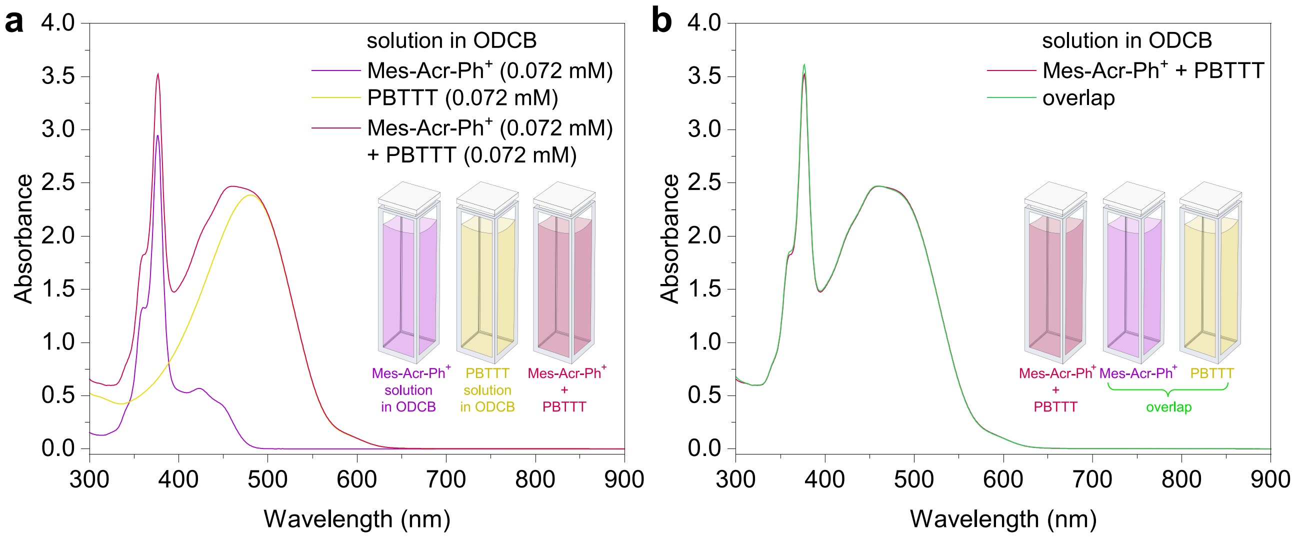

Supplementary Fig. 18 | Photocatalyst-semiconductor interaction. a-b, Absorption spectra of Mes-Acr-Ph$^+$, PBTTT, and their mixture in 1,2-dichlorobenzene (ODCB). Purple curve: Mes-Acr-Ph$^+$; Yellow curve: PBTTT; Crimson curve: mixture of Mes-Acr-Ph$^+$ and PBTTT; Green curve: overlap of Mes-Acr-Ph$^+$ and PBTTT absorptions. Note that only Mes-Acr-Ph$^+$ (BF_4^-) has good solubility in non-polar solvents like ODCB. No observable changes in absorption occur in the mixture, indicating the absence of a significant interaction between the ground state Mes-Acr-Ph$^+$ and PBTTT.

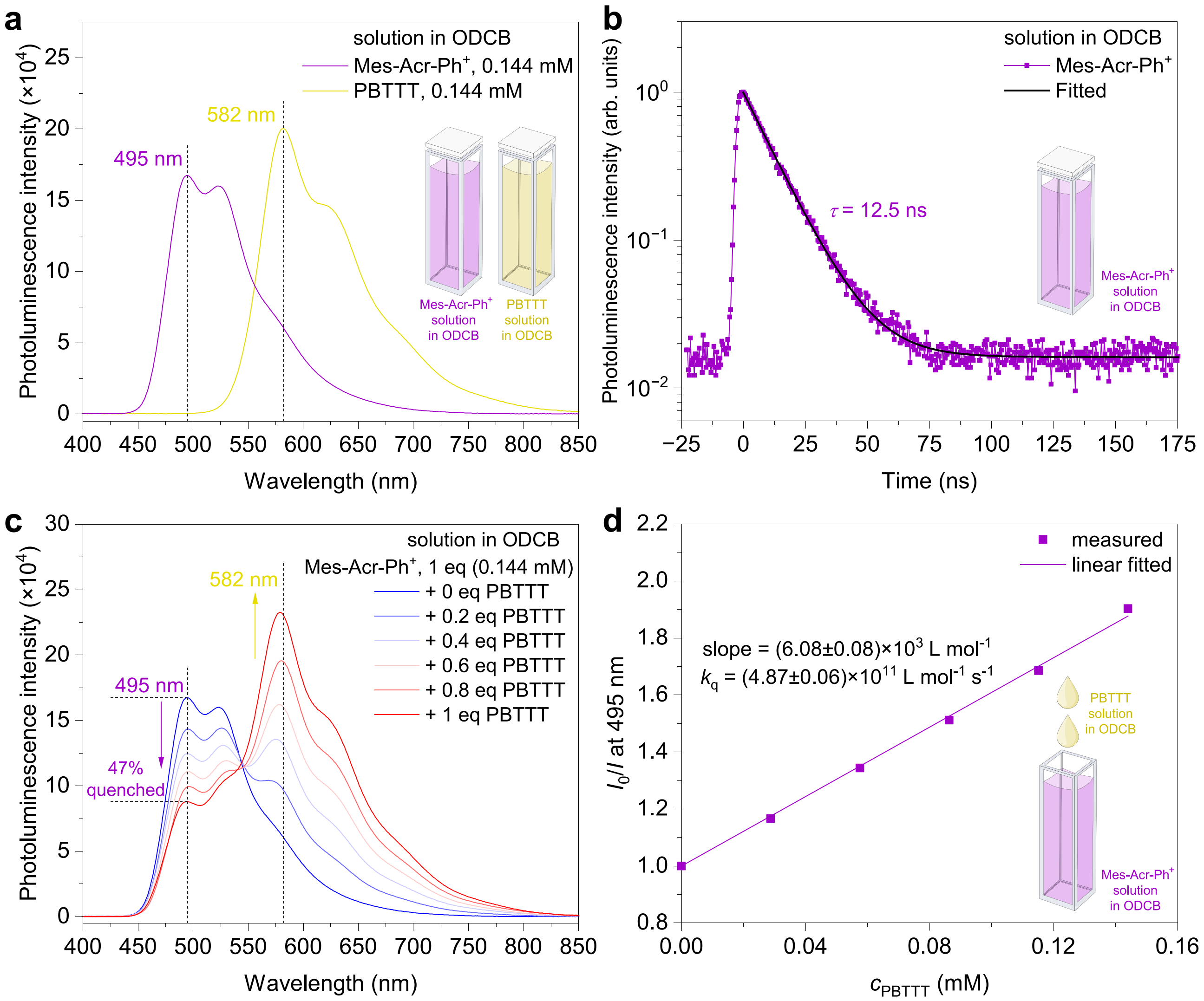

Supplementary Fig. 19 | Stern–Volmer relationship. a, Photoluminescence spectra of Mes-Acr-Ph^+ and PBTTT in ODCB. The excitation wavelength is 400 nm. **b**, Time-resolved photoluminescence spectra of Mes-Acr-Ph^+, showing a long fluorescence lifetime of $\tau_0 = 12.5$ ns. **c**, Fluorescence quenching of 0.144 mM Mes-Acr-Ph^+ by 0 to 1 equivalent of PBTTT. **d**, Stern–Volmer plot of fluorescence quenching of Mes-Acr-Ph^+ by PBTTT. The quenching rate constant ($k_q = 4.9\times10^{11}$ L mol^{-1} s^{-1}) is extracted from the Stern–Volmer relationship: $I/I_0 = 1 + k_q\cdot\tau_0\cdot c_{PBTTT}$, where I_0 and I are the fluorescence intensity of Mes-Acr-Ph^+ without/with quencher PBTTT, and c_{PBTTT} is the concentration of PBTTT. The fluorescence of Mes-Acr-Ph^+ is quenched by 47% using 1 eq of PBTTT, showing a strong interaction between the Mes-Acr-Ph^{+*}'s excited state and the PBTTT's ground state.

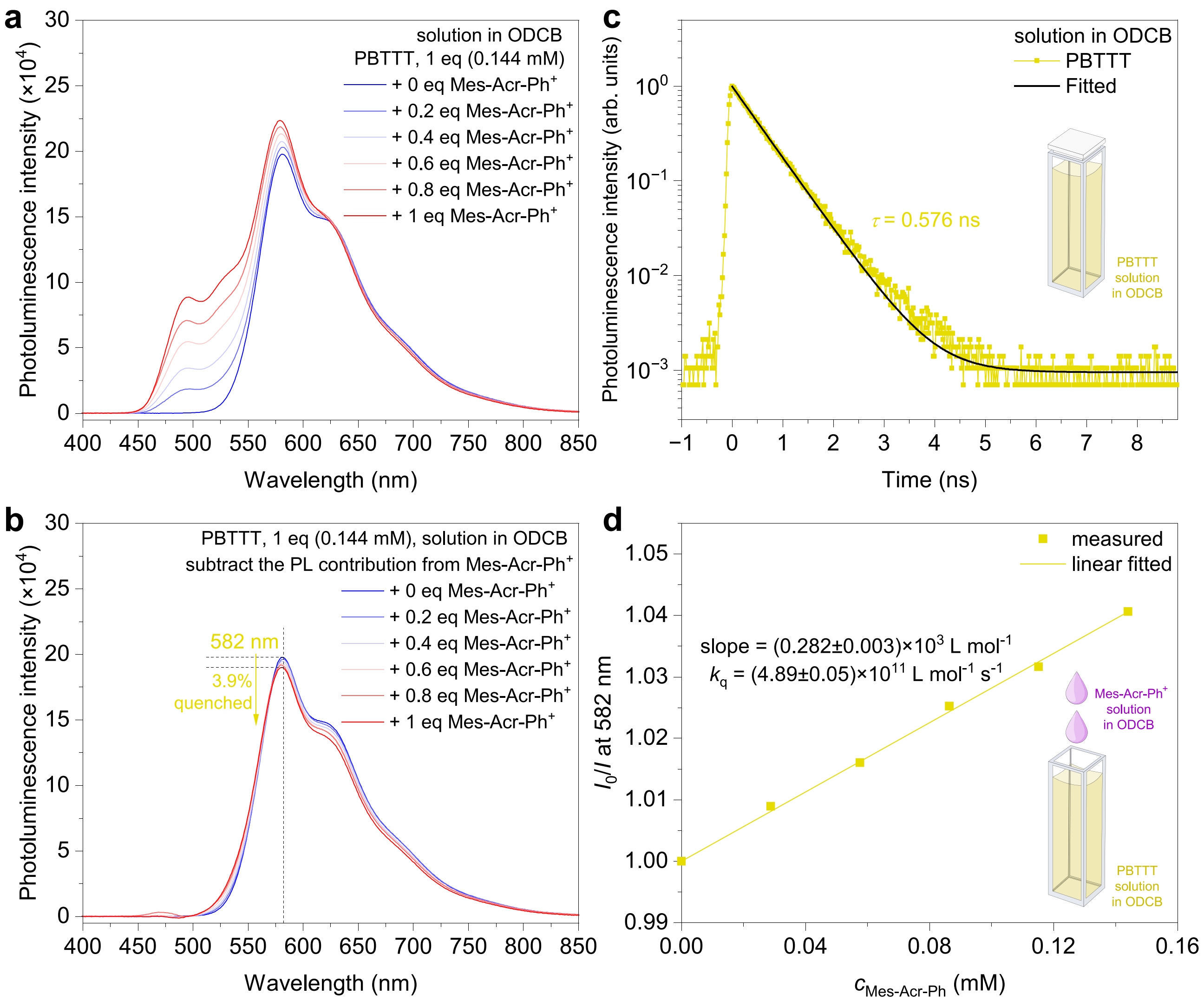

Supplementary Fig. 20 | Stern–Volmer relationship. a, Photoluminescence spectra of PBTTT (1 eq) and Mes-Acr-Ph^+ (0 to 1 eq) in ODCB. The excitation wavelength is 400 nm. **b**, Time-resolved photoluminescence spectra of PBTTT, showing a short fluorescence lifetime of τ_0 = 0.576 ns. **c**, Fluorescence quenching of 0.144 mM Mes-Acr-Ph^+ by 0 to 1 eq PBTTT (subtracted the fluorescence of Mes-Acr-Ph^+). **d**, Stern–Volmer plot of fluorescence quenching of PBTTT by Mes-Acr-Ph^+. The quenching rate constant ($k_q = 4.9\times10^{11}$ L mol^{-1} s^{-1}) is extracted from Stern–Volmer relationship: $I/I_0 = 1 + k_q\cdot\tau_0\cdot c_{Mes\text{-}Acr\text{-}Ph^+}$, where I_0 and I are the fluorescence intensity of PBTTT without/with quencher Mes-Acr-Ph^+, and $c_{Mes\text{-}Acr\text{-}Ph^+}$ is the concentration of Mes-Acr-Ph^+. The fluorescence of PBTTT is quenched by ca. 4% using 1 eq Mes-Acr-Ph^+, showing a weaker interaction between the Mes-Acr-Ph^{+}'s ground state and the $PBTTT^*$'s excited state.

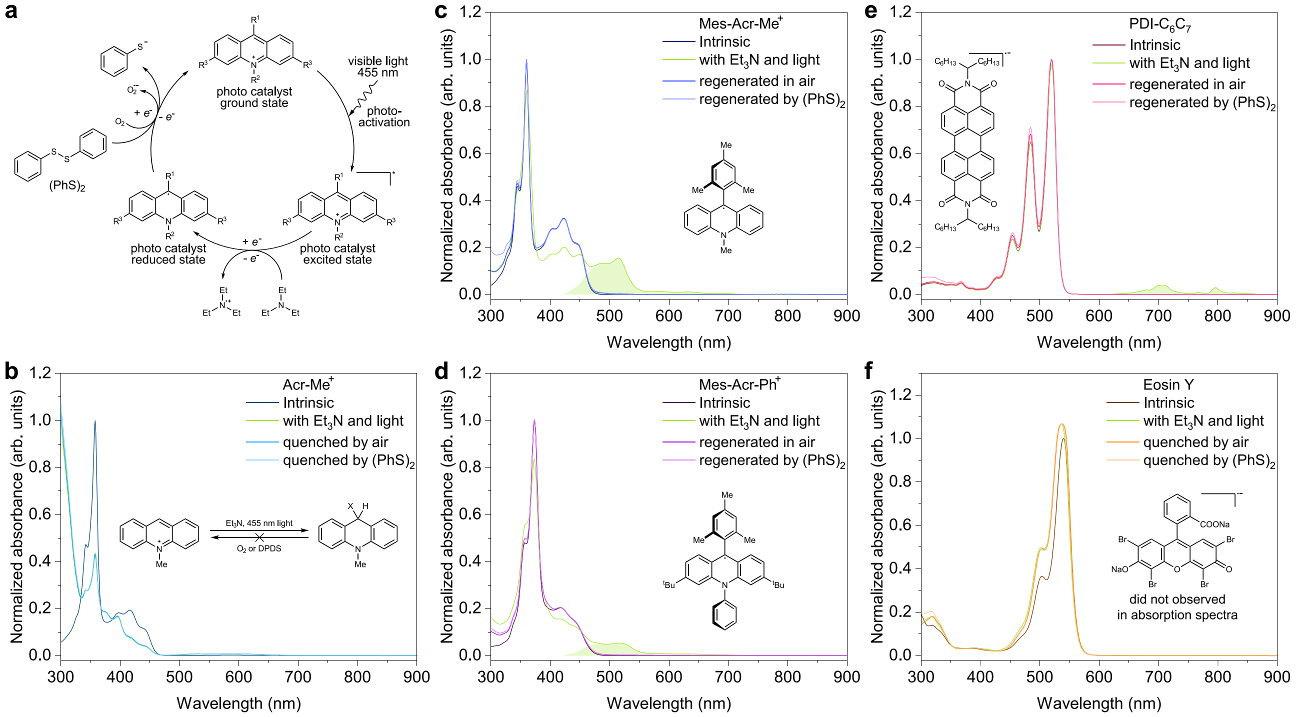

Supplementary Fig. 21 | Photocatalyst reduction and regeneration. a, Schematic diagram of the photocatalyst reduction by the optically transparent reductant triethylamine (Et_3N, 2 eq) and oxidation regeneration by O_2 or $(PhS)_2$. **b-f**, Absorption spectra of the intrinsic photocatalysts, treated with 455 nm light and Et_3N, and quenched (regenerated) by O_2 or $(PhS)_2$ for Acr-Me$^+$ (**b**), Mes-Acr-Me$^+$ (**c**), Mes-Acr-Ph$^+$ (**d**), PDI-C_6C_7 (**e**), and Eosin Y (**f**). Acr-Me$^+$ likely forms irreversible addition products with Et_3N due to the absence of a steric protecting group. Reduction of Eosin Y was not observed from the absorption spectra. Mes-Acr-Me$^+$, Mes-Acr-Ph$^+$ and PDI-C_6C_7 show clear reduced states with triethylamine and oxidative regeneration ability.

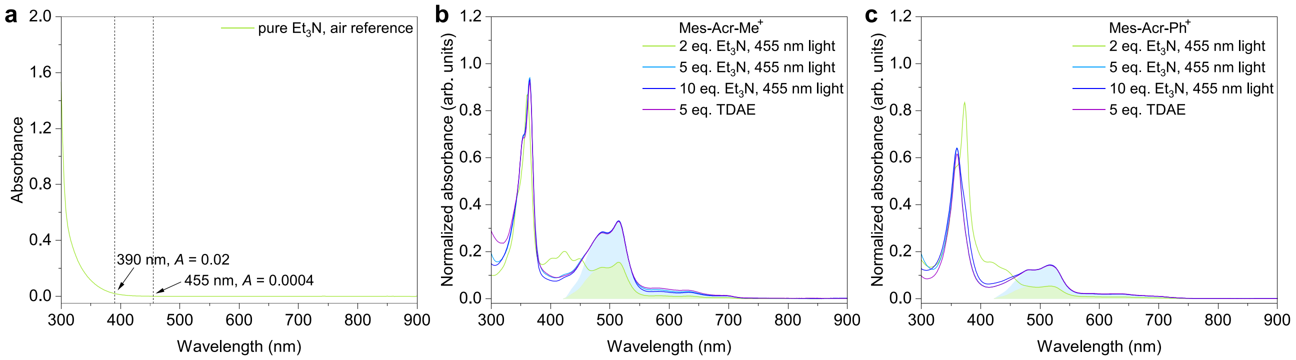

Supplementary Fig. 22 | Photocatalyst reduction. a, Absorption spectra of Et_3N, showing its optical transparency at 455 nm or 390 nm. **b-c**, Absorption spectra of reduced state Mes-Acr-Me$^+$ (**b**) and Mes-Acr-Ph$^+$ (**c**), photo-reduced by 2-10 eq of Et_3N or reduced by tetrakis(dimethylamino)ethylene (TDAE). Et_3N (5-10 eq) can fully convert Mes-Acr-Me$^+$ or Mes-Acr-Ph$^+$ to their reduced states via photoreduction.

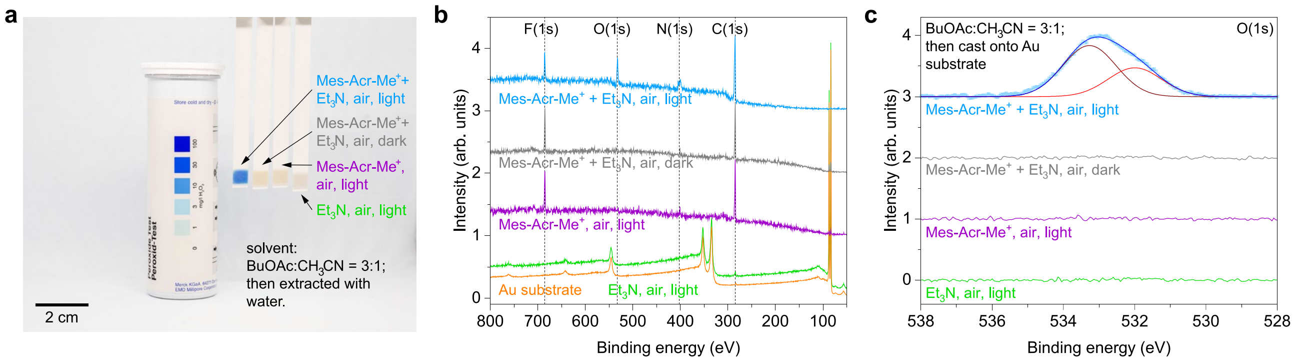

Supplementary Fig. 23 | O_2 reduction during photocatalyst regeneration. a, Photograph of the peroxide colorimetric test strip, showing peroxide formation upon photocatalyst regeneration in air. Mes-Acr-Me^+ (25 µmol) and Et_3N (50 µmol) were dissolved in BuOAc:CH_3CN (3:1, 1 mL) in air, and the solution was irradiated at 455 nm to convert Mes-Acr-Me^+ into Mes-Acr-$Me^•$. The latter is reconverted to Mes-Acr-Me^+ by oxidation with air. Because of the strong yellow color of Mes-Acr-Me^+, a direct colorimetric assay of the photocatalyst solution could not be carried out. We then added 4 mL of deionized water to extract any generated peroxide. The water phase was tested with a peroxide colorimetric test strip (Sigma-Aldrich). Control samples without light (gray), without Et_3N (purple), and without Mes-Acr-Me^+ (green) were also tested. **b**, XPS analysis of the O-containing product formed during the photocatalyst regeneration process: Mes-Acr-Me^+ (BF_4^-) and Et_3N (10 eq) were dissolved in CH_3CN and irradiated at 455 nm in the air for 15 minutes. The solution was then cast onto an Au-coated glass substrate. Notably, none of the photocatalysts (Mes-Acr-Me^+:$BF4^-$), reductant (Et_3N), and solvent (CH_3CN) contain oxygen. Control samples (without photocatalyst: green curve; without Et_3N: purple curve; without light: gray curve; pure Au-coated glass substrate: orange curve) were also tested. **c**, O(1s) XPS spectra display a significant O(1s) signal with two peaks at 532 eV and 533.3 eV.

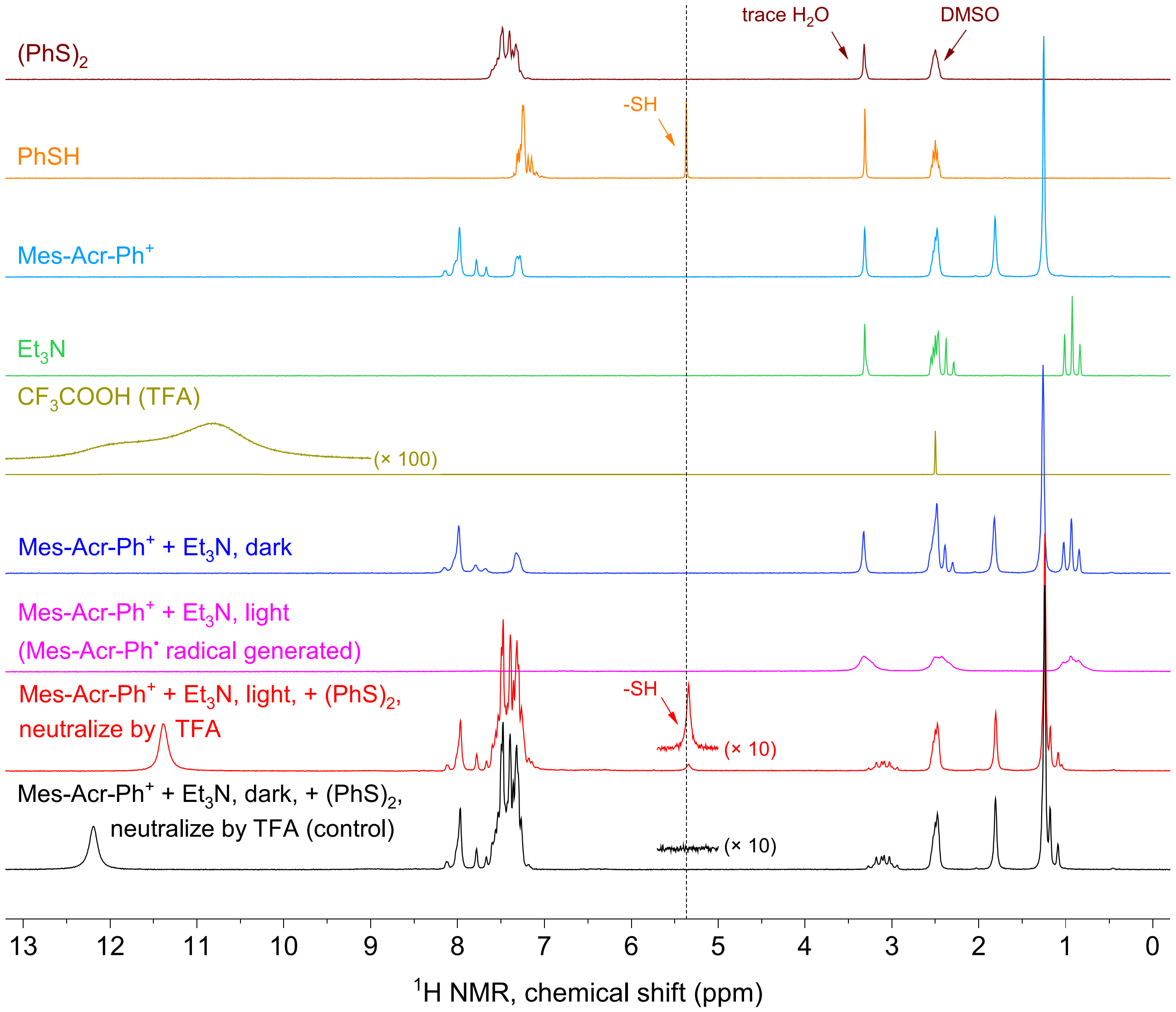

Supplementary Fig. 24 | ^{1}H NMR spectra of $(PhS)_2$ during regeneration of Mes-Acr-Ph$^+$(BF_4^-). Mes-Acr-Ph$^+$ was mixed with 2 eq Et_3N in DMSO-d_6 (deep blue curve), then irradiated at 455 nm to generate Mes-Acr-Ph$^{\bullet}$ (magenta curve). Then, $(PhS)_2$ was added to the solution to regenerate Mes-Acr-Ph$^+$, while trifluoroacetate (TFA) was added to neutralize formed thiophenol anions (red curve). A distinct thiophenol -SH signal was observed, which was not visible in the control experiment (without 455 nm light irradiation, black curve). Additionally, ^{1}H NMR spectra of pure $(PhS)_2$ (maroon curve), thiophenol (orange curve), Mes-Acr-Ph$^+$(BF_4^-) (light blue curve), Et_3N (green curve), and trifluoroacetate (dark yellow curve) in DMSO-d_6 are also presented.

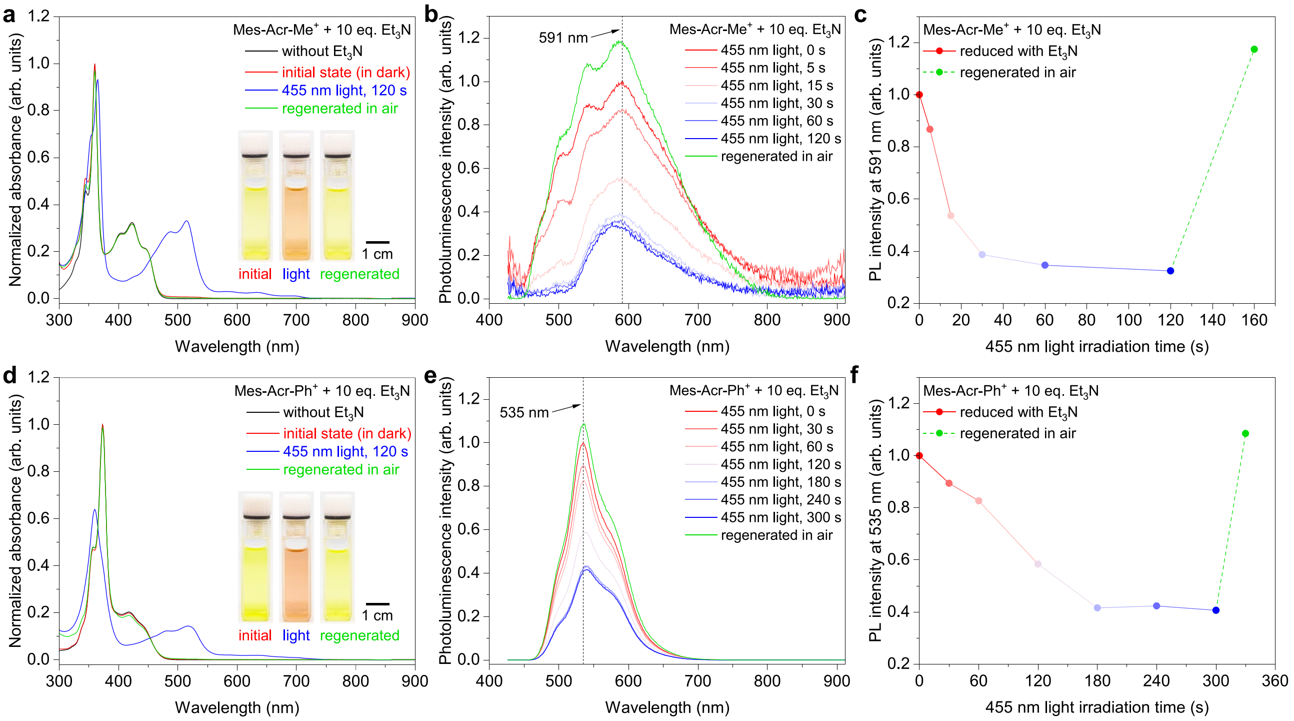

Supplementary Fig. 25 | Photocatalyst reduction and regeneration. a, Absorption spectra of Mes-Acr-Me$^+$/Et_3N solution, in the initial state (in dark), irradiated under 455 nm light, and regenerated in air. Inset: photographs of Mes-Acr-Me$^+$/Et_3N solution in the above three states. See Supplementary Video 2 for the vivid regeneration process. Absorption spectra of pure Mes-Acr-Me$^+$ are also shown for comparison, demonstrating that Et_3N cannot reduce Mes-Acr-Me$^+$ without light. **b**, *In-situ* photoluminescence spectra of Mes-Acr-Me$^+$/Et_3N solution, in the initial state, irradiated under 455 nm light and regenerated in air. **c**, Photoluminescence intensity at 591 nm of the above Mes-Acr-Me$^+$/Et_3N solution. **d,** Absorption spectra of Mes-Acr-Ph$^+$/Et_3N solution, in the initial state (in dark), irradiated under 455 nm light and regenerated in air. Inset: photographs of Mes-Acr-Ph$^+$/Et_3N solution in the above three states. Absorption spectra of pure Mes-Acr-Ph$^+$ are also shown for comparison, demonstrating that Et_3N cannot reduce Mes-Acr-Ph$^+$ without light. **e**, *In-situ* photoluminescence spectra of Mes-Acr-Ph$^+$/Et_3N solution, in the initial state, irradiated under 455 nm light and regenerated in air. **c**, Photoluminescence intensity at 535 nm of the above Mes-Acr-Ph$^+$/Et_3N solution. The photocatalysts Mes-Acr-Me$^+$ and Mes-Acr-Ph$^+$ show excellent photoreduction and oxidation regeneration abilities with optically transparent reductant Et_3N and air. This further proves that the photo-redox of photocatalysts does not require the participation of organic semiconductors, further suggesting that photocatalysis is dominated by the excited state of the photocatalysts.

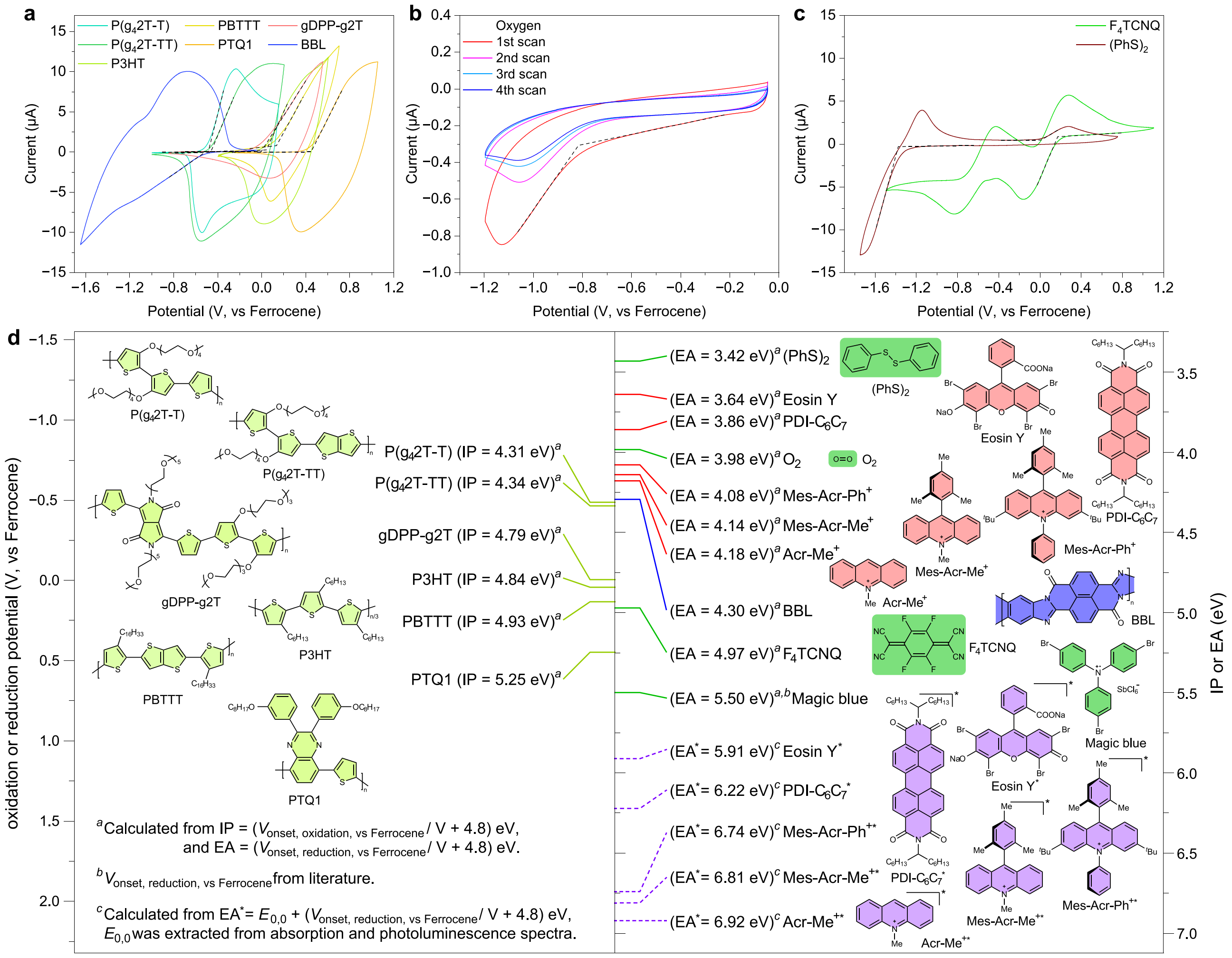

Supplementary Fig. 26 | Redox potentials, ionization potentials, and electron affinities. a-c, Cyclic voltammetry scans in 0.1 M $Bu_4N^+PF_6^-$ BuOAc:CH_3CN (3:1) solution for PTQ1, PBTTT, P3HT, P($g_4$2T-TT), P($g_4$2T-T), gDPP-g2T, and BBL thin films (**a**), O_2 (saturated solution, **b**), and p-dopants F_4TCNQ and $(PhS)_2$ (**c**). The scan speed is 50 mV s^{-1}. **d**, Redox potential; IPs of the organic semiconductors PTQ1, PBTTT, P3HT, P($g_4$2T-TT), P($g_4$2T-T), and gDPP-g2T; EAs of the ground-state and excited-state photocatalysts Acr-Me^+, Mes-Acr-Me^+, Mes-Acr-Ph^+, PDI-C_6C_7, and Eosin Y; EAs of the p-dopant O_2, F_4TCNQ, $(PhS)_2$, and magic blue[2-4]; EA of the n-type polymer BBL.

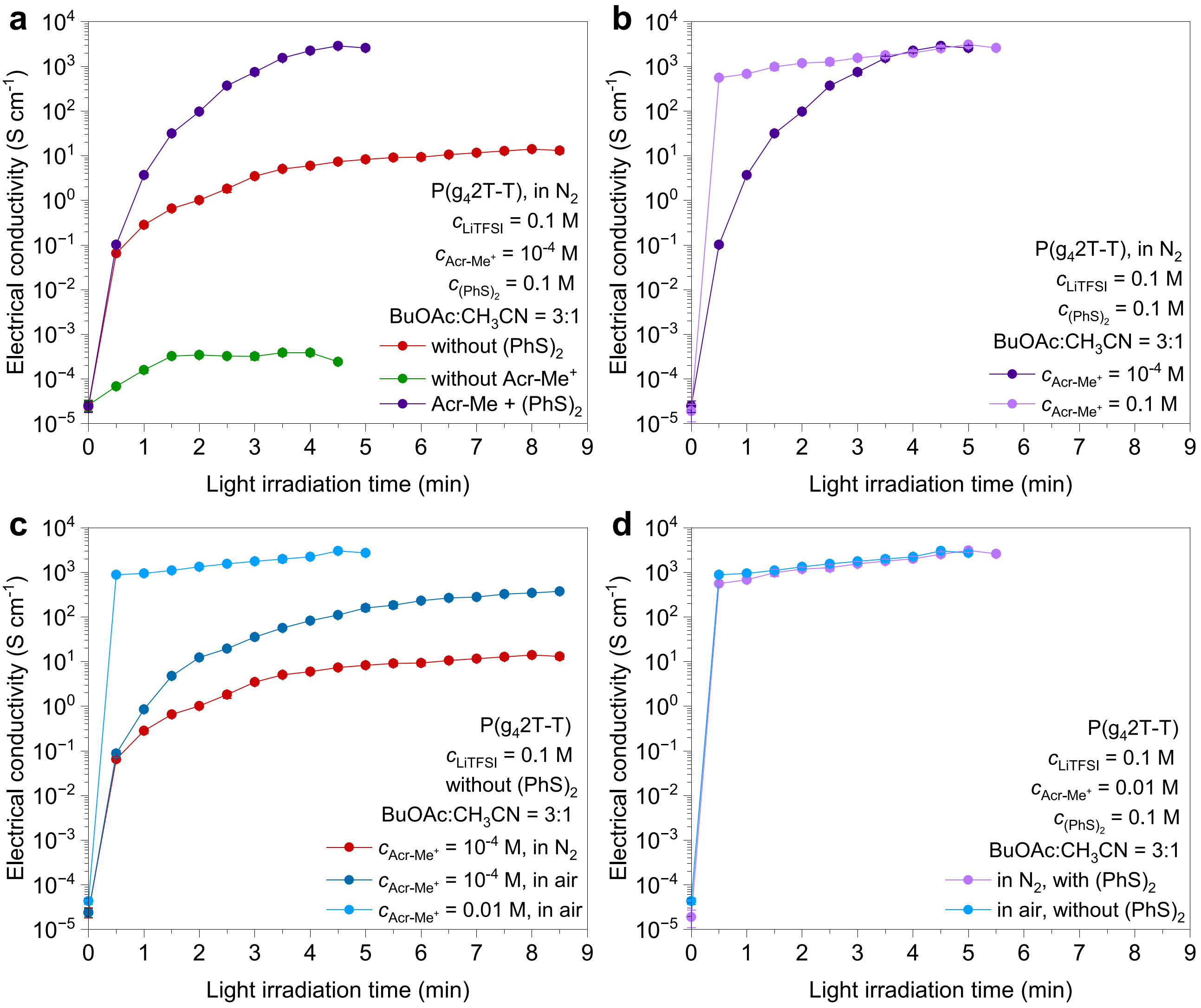

Supplementary Fig. 27 | Doping with weak p-dopant $(PhS)_2$. a, Electrical conductivity of doped $P(g_42T\text{-}T)$ thin films in the absence of p-dopant (diphenyl disulfide, $(PhS)_2$) or photocatalyst (Acr-Me^+), showing that both $(PhS)_2$ and Acr-Me^+ are necessary for p-doping. **b,** Electrical conductivity of doped $P(g_42T\text{-}T)$ thin films in the presence of $(PhS)_2$ with low or high photocatalyst concentration, showing that the doped $P(g_42T\text{-}T)$ shows a consistent trend in the maximum conductivity with a different photocatalyst concentration used. High photocatalyst concentration leads to faster doping. **c,** Electrical conductivity of doped $P(g_42T\text{-}T)$ films with/without O_2. **d,** Comparison of the electrical conductivity of doped $P(g_42T\text{-}T)$ thin films with different p-dopant ($(PhS)_2$ and O_2), showing that O_2 and $(PhS)_2$ show very similar effects in photocatalytic doping at high photocatalyst concentration.

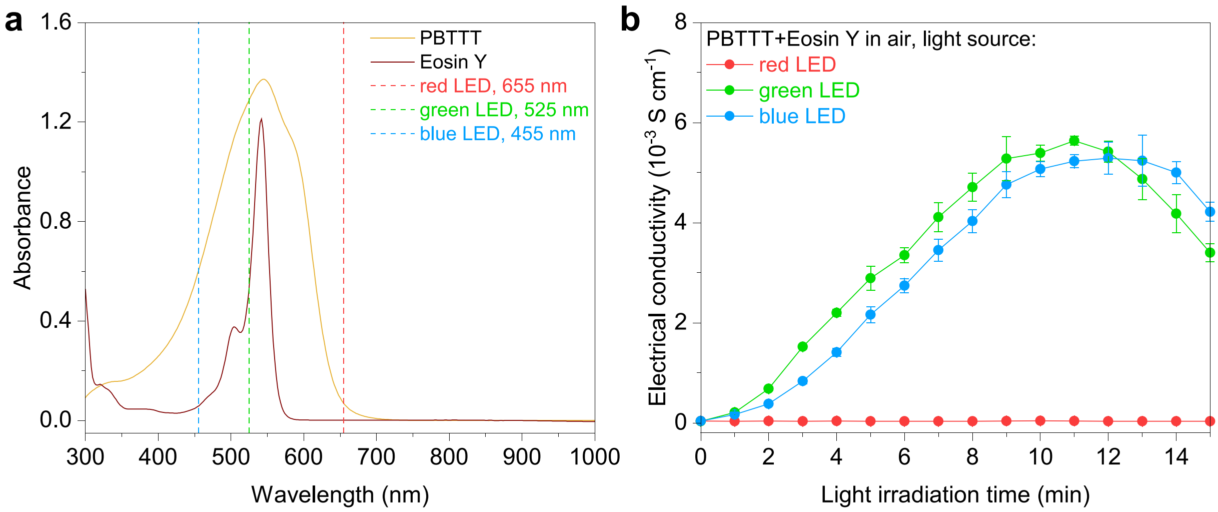

Supplementary Fig. 28 | Effect of excitation wavelength. **a**, Absorption spectra of Eosin Y and PBTTT. **b**, Electrical conductivity of PBTTT photocatalytically p-doped with Eosin Y in air under blue (455 nm), green (525 nm), and red (655 nm) light sources. Note that while PBTTT can be excited by all three light sources, Eosin Y can only be excited by green and blue lights.

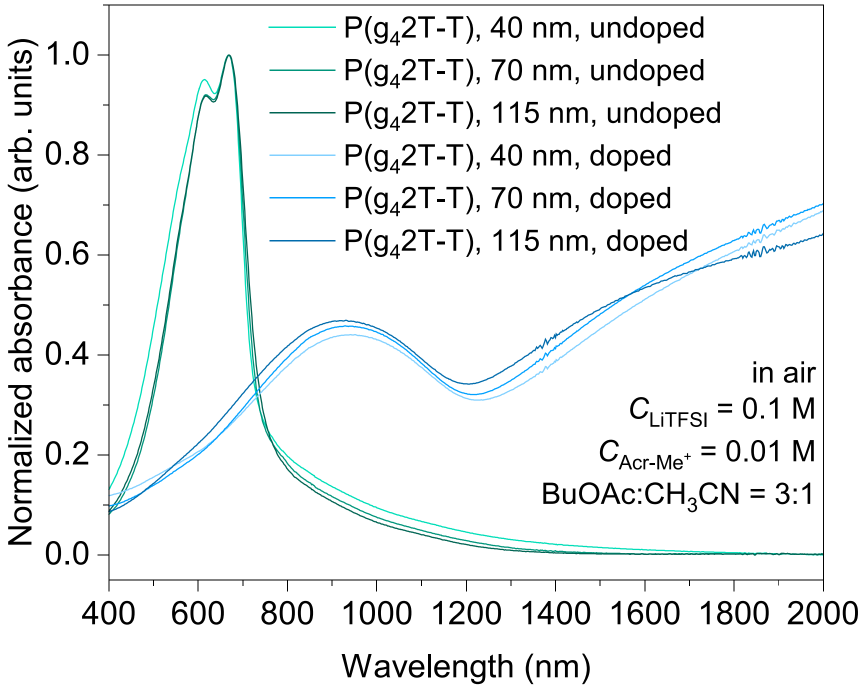

Supplementary Fig. 29 | Bulk doping. Absorption spectra of undoped and doped P($g_4$2T-T) thin films with various thicknesses. All organic semiconductors show clear polaronic absorption, underlining the generality of photocatalytic doping. The P($g_4$2T-T) films with various thicknesses show consistent polaronic absorption intensity, further proving that photocatalytic doping is a bulk effect.

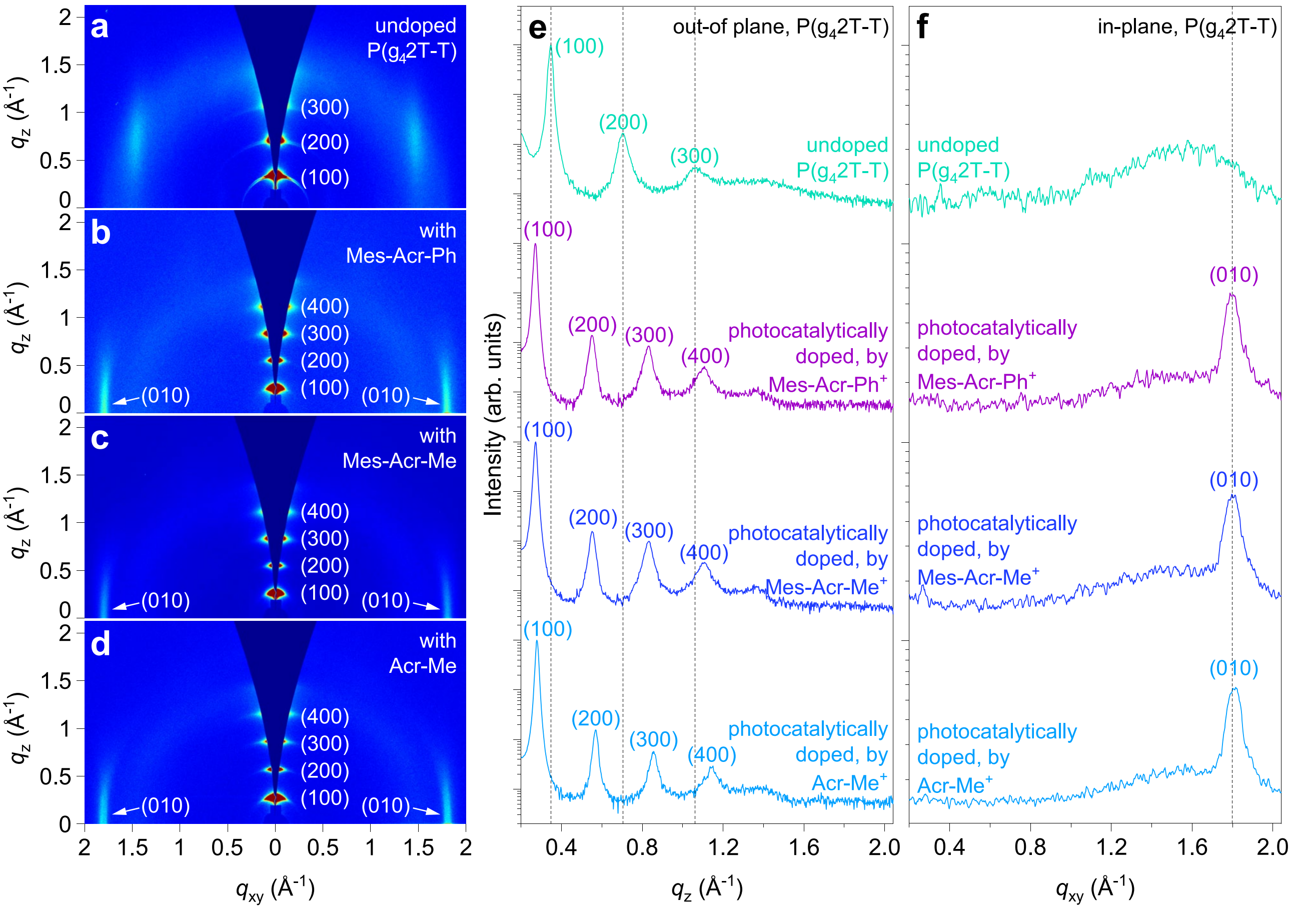

Supplementary Fig. 30 | GIWAXS analysis of P(g42T-T). a-d, 2D GIWAXS patterns of undoped P(g42T-T) (**a**) and doped P(g42T-T) thin films photocatalyzed by different photocatalysts Mes-Acr-Ph$^+$ (**b**), Mes-Acr-Me$^+$ (**c**), and Acr-Me$^+$ (**d**). **e-f,** Out-of-plane (**e**) and in-plane (**f**) GIWAXS line cuts of undoped and doped P(g42T-T) thin films.

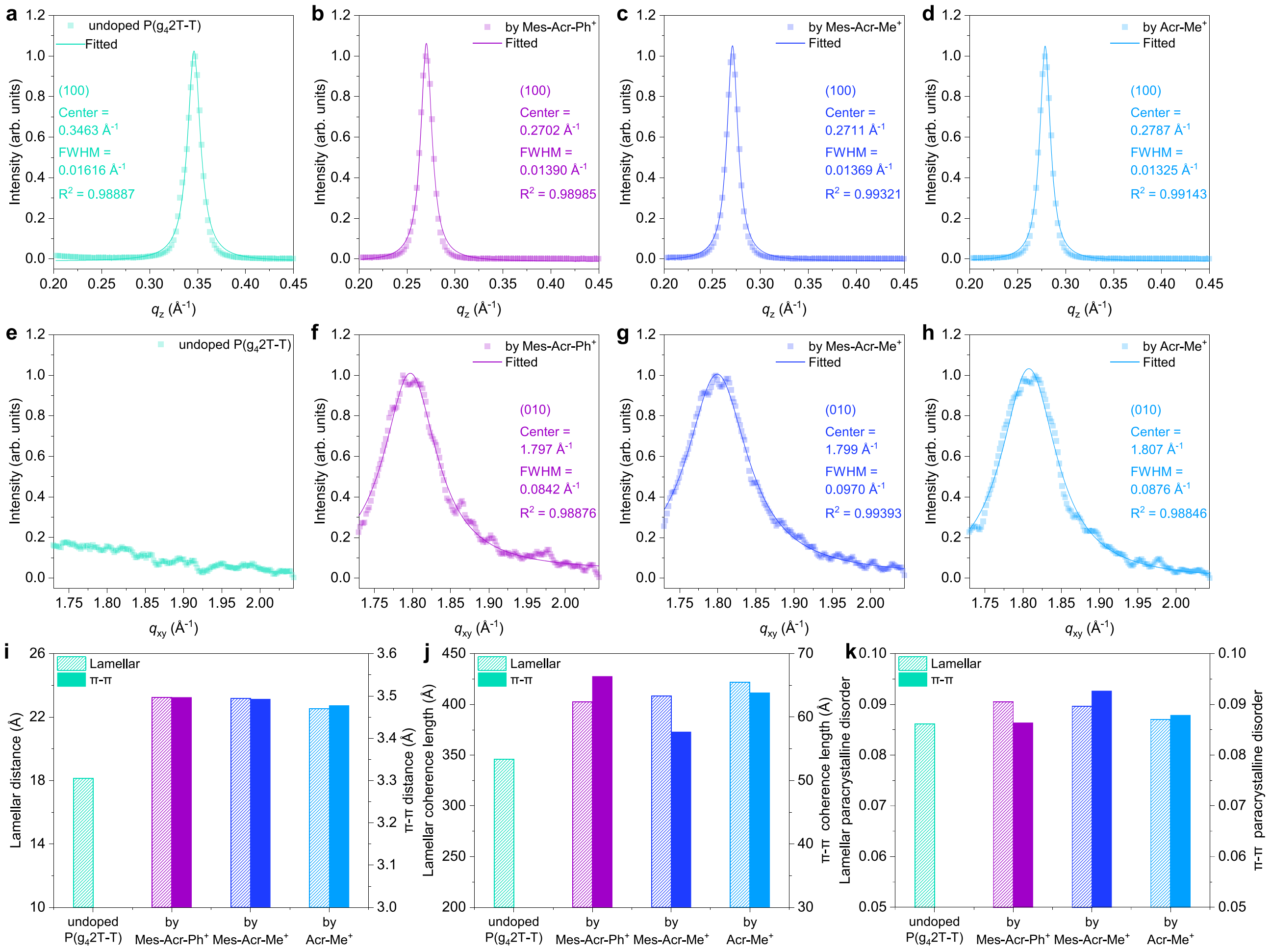

Supplementary Fig. 31 | Lamellar packing and π-π stacking of P(g$_4$2T-T). a-d,** Lamellar packing diffraction peak analysis of undoped P(g$_4$2T-T) (**a**) and doped P(g$_4$2T-T) thin films photocatalyzed by Mes-Acr-Ph$^+$ (b**), Mes-Acr-Me$^+$ (**c**), and Acr-Me$^+$ (**d**). **e-h,** π-π stacking diffraction peak analysis of undoped P(g$_4$2T-T) (**e**) and doped P(g$_4$2T-T) thin films photocatalyzed by Mes-Acr-Ph$^+$ (**f**), Mes-Acr-Me$^+$ (**g**), and Acr-Me$^+$ (**h**). **i-k,** Packing distances (**i**), coherence length (**j**), and paracrystalline disorder (**k**) of lamellar packing and π-π stacking of P(g$_4$2T-T). The π-π stacking diffraction could not be observed in undoped P(g$_4$2T-T), while photocatalytically doped P(g$_4$2T-T) showed clear π-π stacking diffractions. Photocatalytically doped P(g$_4$2T-T) shows a longer lamellar packing distance than undoped P(g$_4$2T-T).

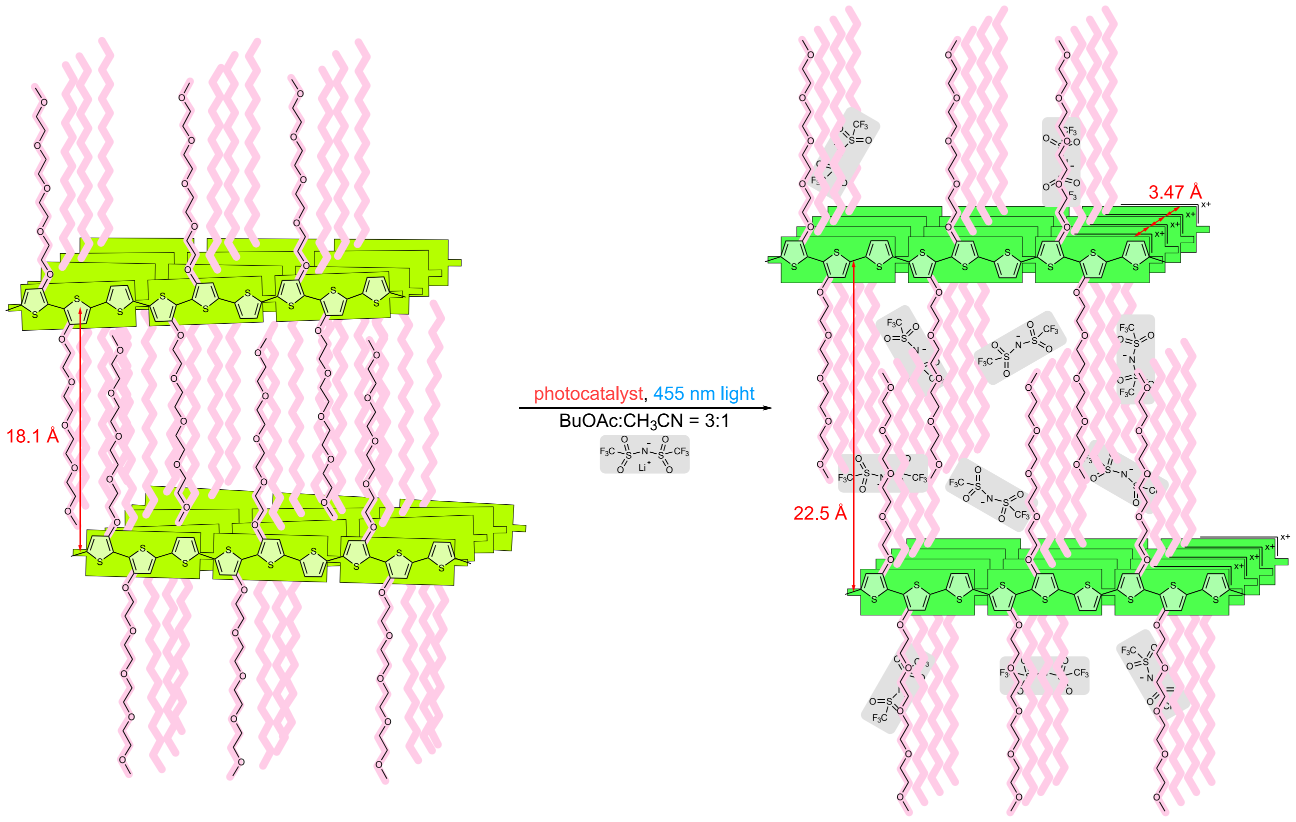

Supplementary Fig. 32 | Molecular packing in photocatalytic doping. Schematic diagram of undoped and photocatalytically doped P(g42T-T) thin films. π-π stacking diffraction could not be observed in undoped P(g42T-T), while photocatalytically doped P(g42T-T) showed clear π-π stacking diffractions. Furthermore, photocatalytically doped P(g42T-T) shows a longer lamellar packing distance than undoped P(g42T-T). This implies that the counterions could be located in the side chain packing region of doped P(g42T-T).

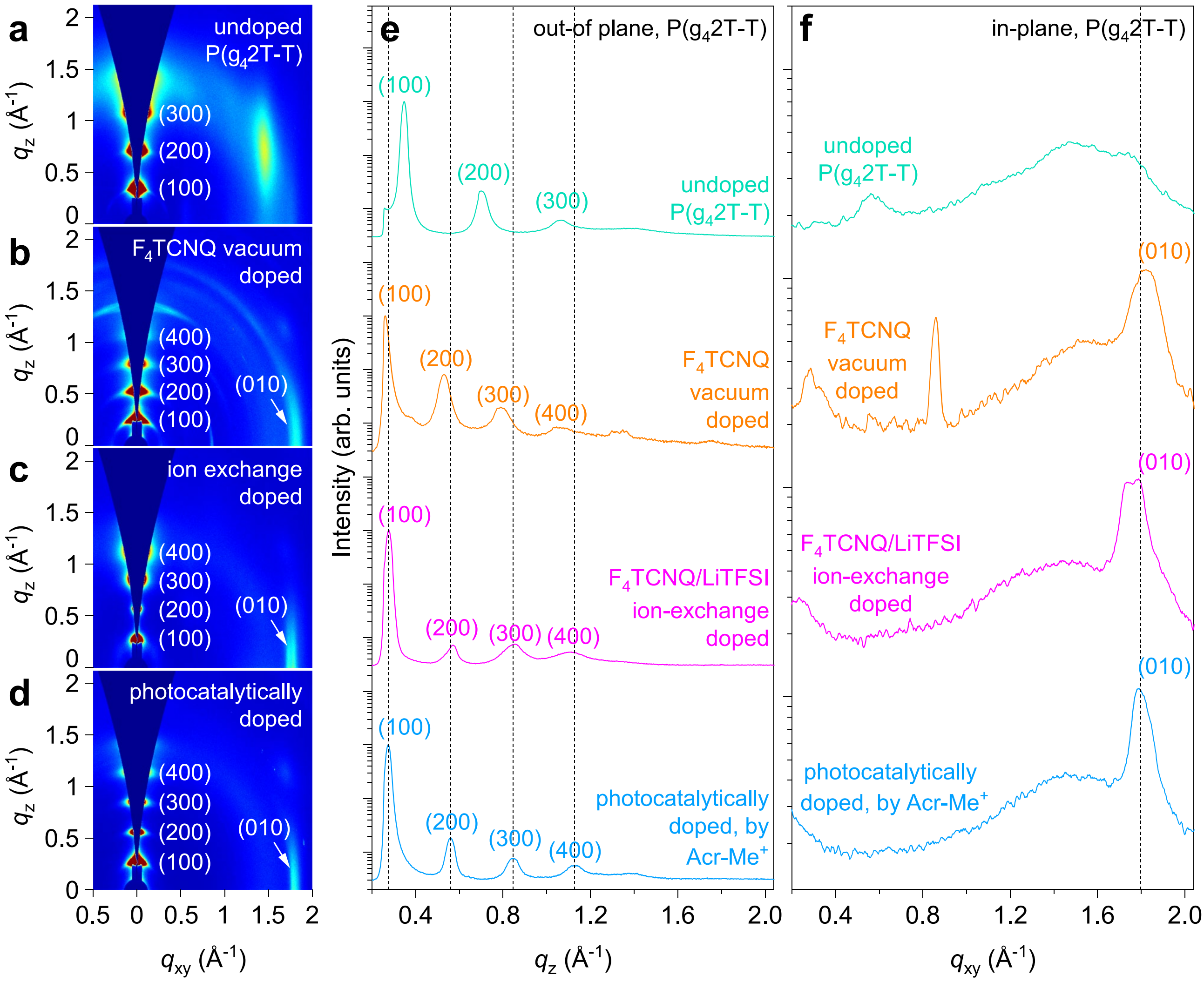

Supplementary Fig. 33 | GIWAXS patterns and line cuts of photocatalytically p-doped P($g_4$2T-T) and comparison with other doping methods. a-d, 2D GIWAXS patterns of undoped P($g_4$2T-T) (**a**), F_4TCNQ vacuum doped P($g_4$2T-T) (**b**), F_4TCNQ/LiTFSI ion exchange doped P($g_4$2T-T) (**c**) and Acr-Me$^+$/air photocatalyzed doped P($g_4$2T-T) (**d**) thin films. **e-f**, Out-of-plane (**e**) and in-plane (**f**) GIWAXS line cuts of undoped and doped P($g_4$2T-T) thin films.

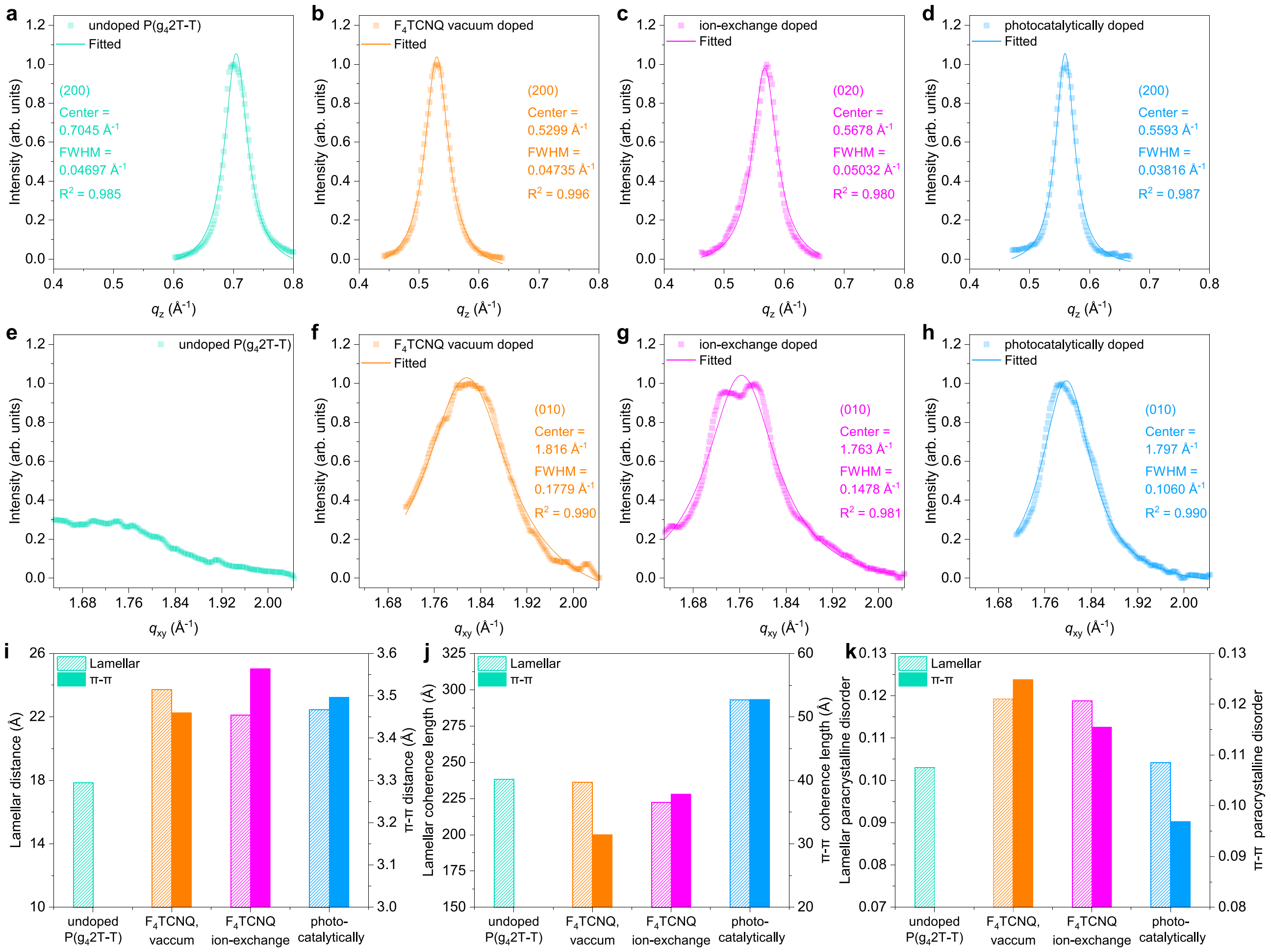

Supplementary Fig. 34 | Crystallinity of P(g42T-T) for different doping methods. a-d, Lamellar packing diffraction peak analysis of undoped P(g42T-T) (**a**), F_4TCNQ vacuum doped P(g42T-T) (**b**), F_4TCNQ/LiTFSI anion exchanging doped P(g42T-T) (**c**) and Acr-Me^+/air photocatalyzed doped P(g42T-T) (**d**) thin films. **e-h**, π-π stacking diffraction peak analysis of undoped P(g42T-T) (**e**), F_4TCNQ vacuum doped P(g42T-T) (**f**), F_4TCNQ/LiTFSI ion exchange doped P(g42T-T) (**g**) and Acr-Me^+/air photocatalyzed doped P(g42T-T) (**h**) thin films. **i-k**, Packing distances (**i**), coherence length (**j**), and paracrystalline disorder (**k**) of lamellar packing and π-π stacking of P(g42T-T) films. Compared to the other doping methods, the photocatalytic doping method yields doped PBTTT films with the highest crystallinity. Note that the values in **i-k,** extracted from the GIWAXS line cuts of Supplementary Fig. 33, differ slightly from those reported in Supplementary Fig. 31, as these measurements were conducted during a different beamtime session.

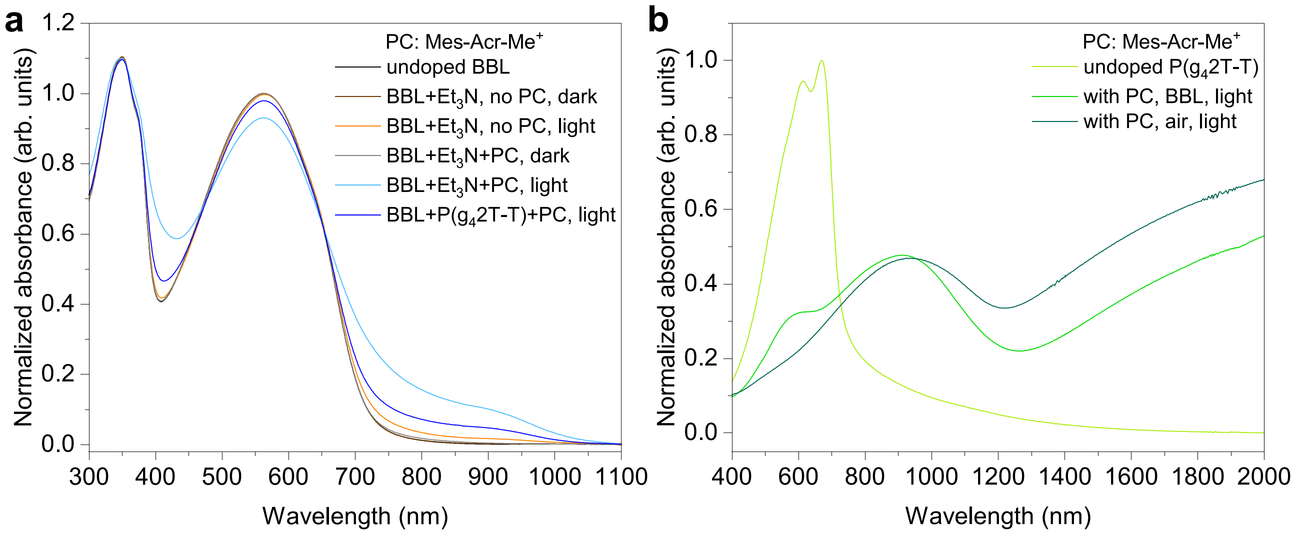

Supplementary Fig. 35 | Photocatalytic n-doping. a, Absorption spectra of undoped BBL, triethylamine and P($g_4$2T-T) doped (photocatalyzed by Mes-Acr-Me^+) BBL thin films. **b,** Absorption spectra of undoped P($g_4$2T-T), BBL doped (photocatalyzed by Mes-Acr-Me^+) and oxygen doped P($g_4$2T-T) thin films.

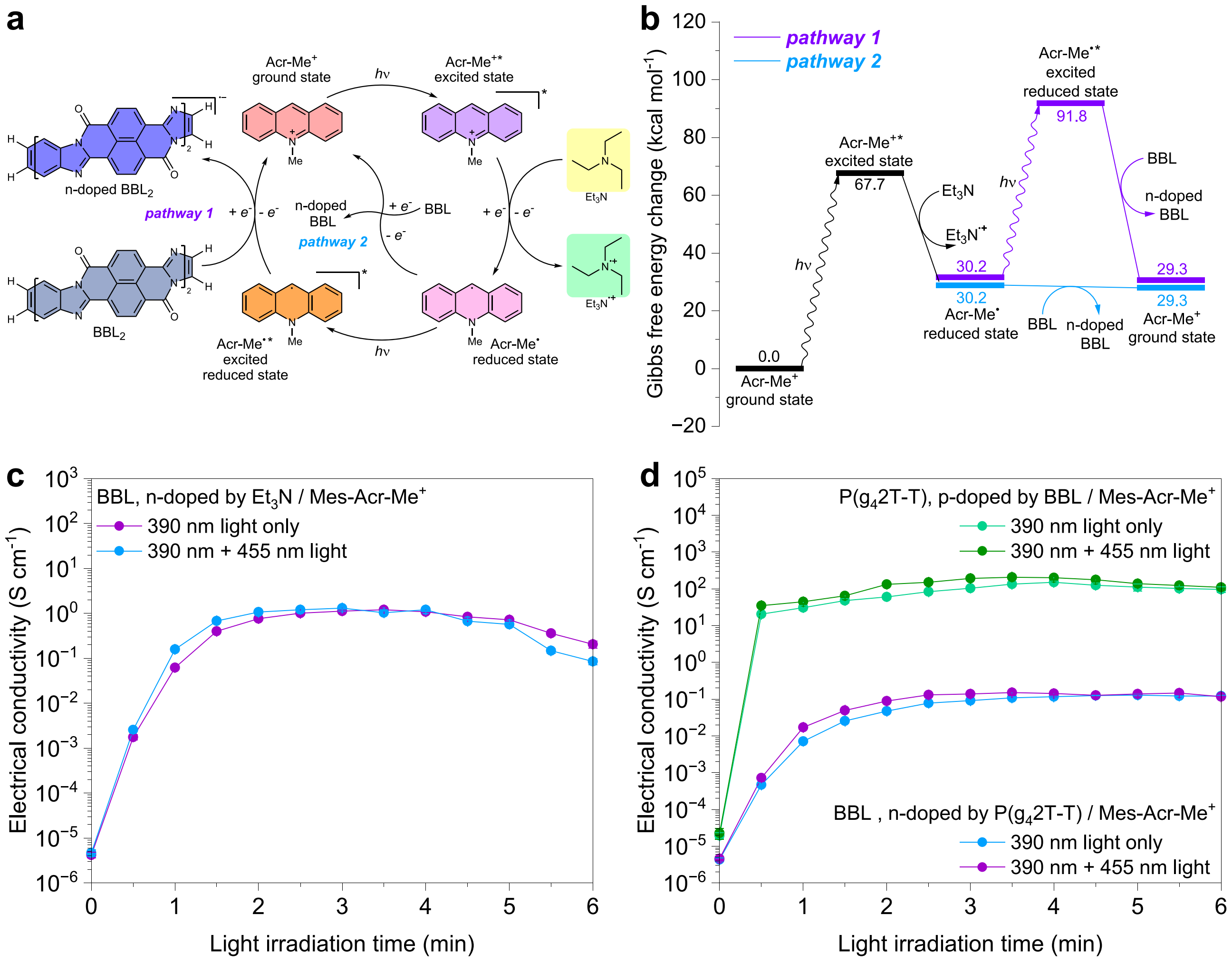

Supplementary Fig. 36 | Photocatalytic n-doping mechanisms. a, Photocatalytic cycle showing two alternative pathways for the n-doping of BBL by Acr-Me$^+$-based photocatalyst and Et$_3$N. **b**, DFT-calculated Gibbs free energy change for pathway 1 and pathway 2. **c**, Electrical conductivity of BBL films photocatalytically doped by Mes-Acr-Me$^+$ and Et$_3$N under 390 nm and 455 nm + 390 nm light irradiation. **c**, Electrical conductivity of BBL and P(g$_4$2T-T) films photocatalytically doped by Mes-Acr-Me$^+$ under 390 nm and 455 nm + 390 nm light irradiation. 390 nm and 455 nm light exposure powers were set to both 50 mW cm^{-2}.

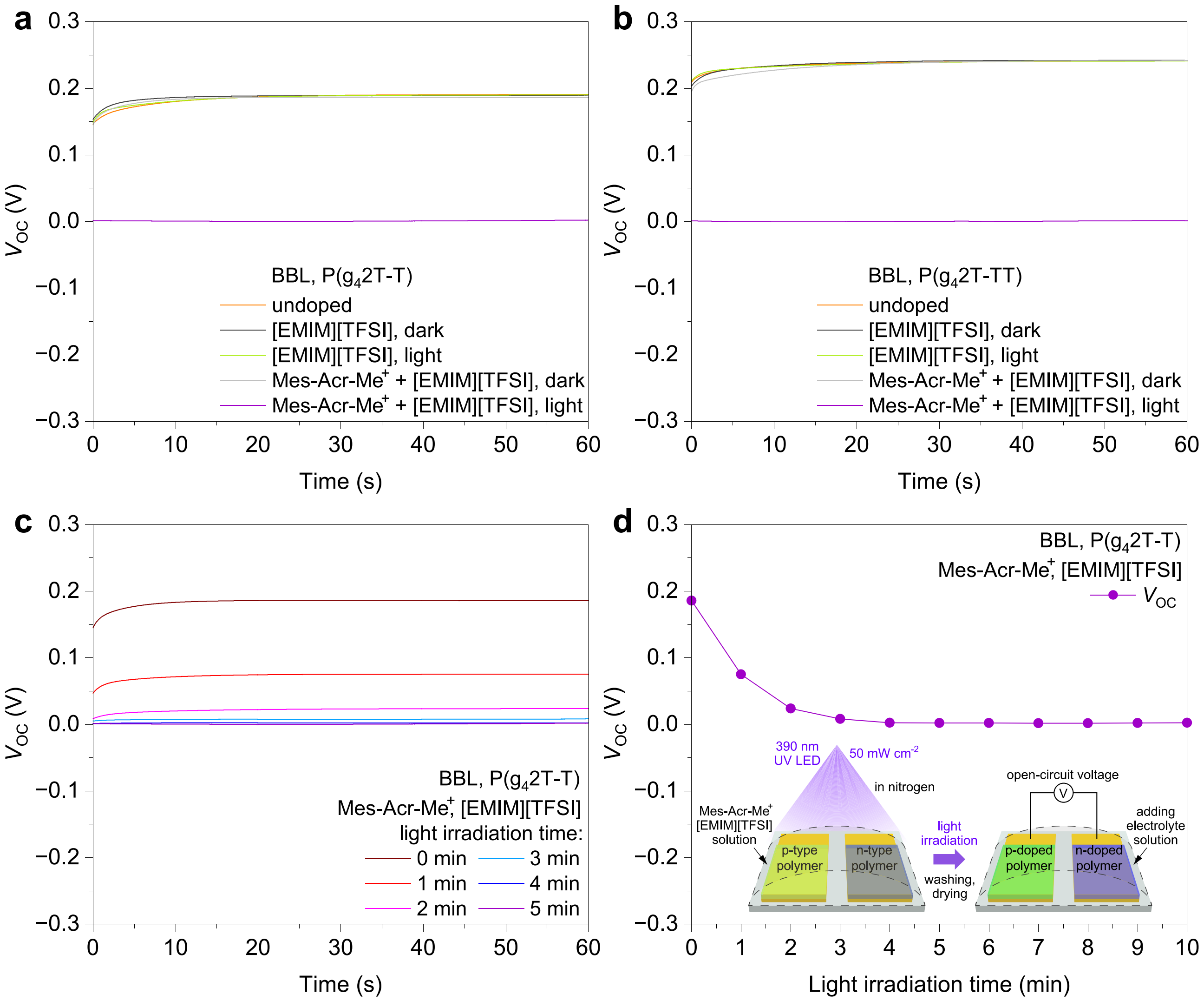

Supplementary Fig. 37 | Fermi level alignment during simultaneous p/n-doping. a-b, Open-circuit voltage measurement during simultaneous p/n-doping between BBL and P($g_4$2T-T) (**a**) or P($g_4$2T-TT) (**b**). The p-type and n-type polymers were deposited to two separate gold electrodes coated on a glass substrate. The photocatalyst solution (0.01 M Mes-Acr-Me^+ and 0.1 M [EMIM][TFSI] in BuOAc:CH_3CN = 3:1) was added under nitrogen to cover the p-/n-type polymers, followed by light irradiation at 390 nm for 5 min. The polymer films were washed with clean solvent and dried with nitrogen flow. The electrolyte solution (0.1 M [EMIM][TFSI] in BuOAc:CH_3CN = 3:1) was added to connect p-/n-type polymers, and the open-circuit voltage (V_{OC}) was measured under nitrogen. Control experiments were also performed as follows: [EMIM][TFSI] in dark and light, Mes-Acr-Me^+ + [EMIM][TFSI] in dark. Before doping, the V_{OC} between BBL and P($g_4$2T-T) is about 0.19 V [0.24 V between BBL and P($g_4$2T-TT)]. Upon photocatalytic p/n-doping, the V_{OC} drops to 0 V. **c**, Open-circuit voltage measurements for BBL and P($g_4$2T-T) under different light irradiation times. **d**, Light irradiation time dependent open-circuit voltage for BBL and P($g_4$2T-T).

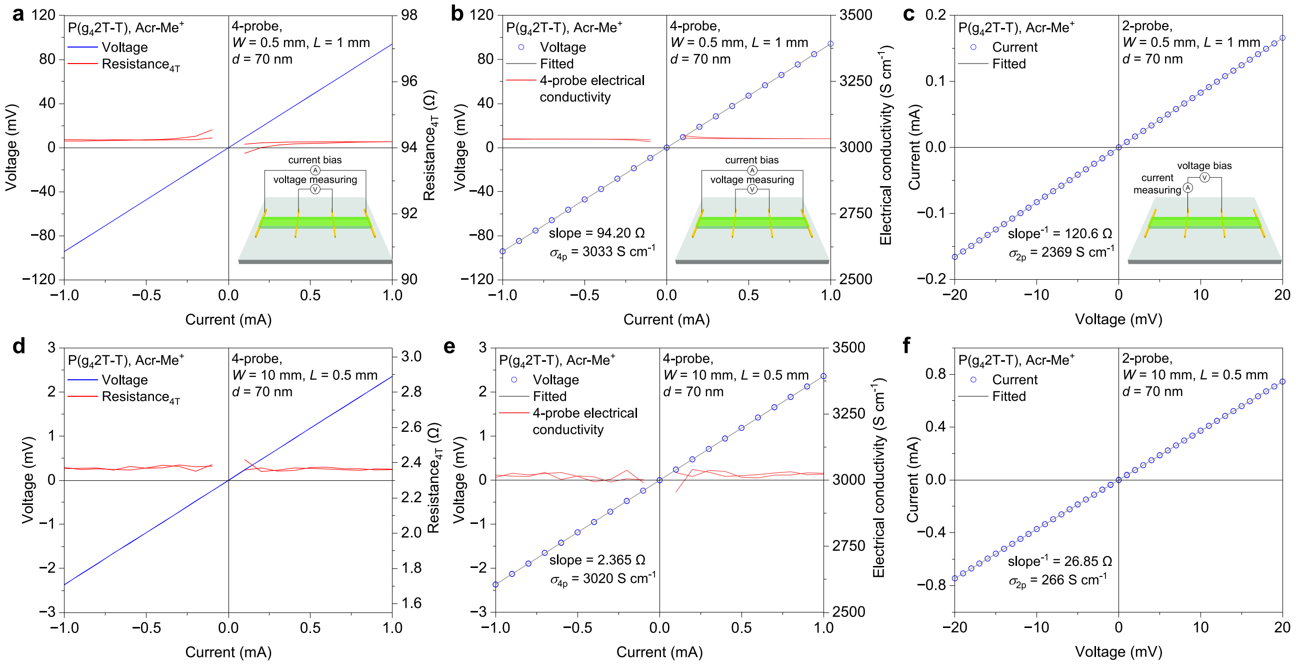

Supplementary Fig. 38 | 4-probe and 2-probe electrical conductivities. a-c, 4-probe conductivity (**a**, **b**) and 2-probe conductivity (**c**) measurements for P(g42T-T) (photocatalytically p-doped by Acr-Me^{+}/air) with channel width W = 0.5 mm and channel length L = 1 mm (W/L = 0.5), showing 4-probe conductivity of ~3000 S cm^{-1} and 2-probe conductivity of ~2370 S cm^{-1}. **d-f**, 4-probe conductivity (**d**, **e**) and 2-probe conductivity (**f**) measurements for P(g42T-T) (p-doped by Acr-Me^{+}/air) with channel width W = 10 mm and channel length L = 0.5 mm (W/L = 20), showing 4-probe conductivity of ~3000 S cm^{-1} and 2-probe conductivity of ~270 S cm^{-1}.

Supplementary Table 1. Maximum electrical conductivity of the organic semiconductors P($g_4$2T-T), P($g_4$2T-TT), P3HT, PBTTT, PTQ1, and gDPP-g2T photocatalytically doped by Acr-Me, Mes-Acr-Me, Mes-Acr-Ph, PDI-C_6C_7 and Eosin Y in air.

Photocatalyst	P($g_4$2T-T) conductivity (S cm^{-1})	P($g_4$2T-TT) conductivity (S cm^{-1})	P3HT conductivity (S cm^{-1})	PBTTT conductivity (S cm^{-1})	PTQ1 conductivity (S cm^{-1})	gDPP-g2T conductivity (S cm^{-1})
undoped	4.32 ± 0.70 $\times10^{-5}$	2.23 ± 0.39 $\times10^{-5}$	2.35 ± 0.04 $\times10^{-5}$	3.77 ± 0.66 $\times10^{-5}$	6.91 ± 1.17 $\times10^{-7}$	3.74 ± 1.21 $\times10^{-5}$
Acr-Me	3011 ± 335	308 ± 17	0.152 ± 0.010	760 ± 6	5.45 ± 0.47 $\times10^{-4}$	158 ± 7
Mes-Acr-Me	1331 ± 128	252 ± 12	0.139 ± 0.001	364 ± 13	3.22 ± 0.19 $\times10^{-4}$	23.8 ± 2.1
Mes-Acr-Ph	1073 ± 170	235 ± 8	0.135 ± 0.005	227 ± 4	3.53 ± 0.24 $\times10^{-4}$	14.9 ± 2.4
PDI-C_6C_7	205 ± 9	61.4 ± 0.3	5.36 ± 0.07 $\times10^{-4}$	1.52 ± 0.04 $\times10^{-2}$	4.35 ± 0.02 $\times10^{-6}$	0.135 ± 0.001
Eosin Y	34.7 ± 2.4	11.3 ± 0.4	2.35 ± 0.04 $\times10^{-5}$	5.29 ± 0.32 $\times10^{-3}$	3.45 ± 0.04 $\times10^{-6}$	0.768 ± 0.026

Supplementary References

1 Romero, N. A. & Nicewicz, D. A. Mechanistic Insight into the Photoredox Catalysis of Anti-Markovnikov Alkene Hydrofunctionalization Reactions. *J. Am. Chem. Soc.* **136**, 17024-17035, (2014).

2 Connelly, N. G. & Geiger, W. E. Chemical redox agents for organometallic chemistry. *Chem. Rev.* **96**, 877-910, (1996).

3 Hofmann, A. I. *et al.* Chemical doping of conjugated polymers with the strong oxidant magic blue. *Adv. Electron. Mater.* **6**, 2000249, (2020).

4 Zhang, S. *et al.* Controllable, wide-ranging n-doping and p-doping of monolayer group 6 transition-metal disulfides and diselenides. *Adv. Mater.* **30**, 1802991, (2018).
